# Global chromatin organizer SATB1 acts as a context-dependent regulator of the Wnt/Wg target genes

**DOI:** 10.1038/s41598-021-81324-2

**Published:** 2021-02-09

**Authors:** Praveena L. Ramanujam, Sonam Mehrotra, Ram Parikshan Kumar, Shreekant Verma, Girish Deshpande, Rakesh K. Mishra, Sanjeev Galande

**Affiliations:** 1 Department of Biology, Centre of Excellence in Epigenetics, Indian Institute of Science Education and Research, Dr. Homi Bhabha Road, Pune, 411008 India; 2 Advanced Centre for Treatment, Research and Education in Cancer, Kharghar, Mumbai India; 3 Centre for Cellular and Molecular Biology, Hyderabad, India; 4 Department of Molecular Biology, Princeton University, Princeton, NJ 08540 USA

**Keywords:** Computational biology and bioinformatics, Developmental biology, Molecular biology

## Abstract

Special AT-rich binding protein-1 (SATB1) integrates higher-order chromatin architecture with gene regulation, thereby regulating multiple signaling pathways. In mammalian cells SATB1 directly interacts with β-catenin and regulates the expression of Wnt targets by binding to their promoters. Whether SATB1 regulates Wnt/wg signaling by recruitment of β-catenin and/or its interactions with other components remains elusive. Since Wnt/Wg signaling is conserved from invertebrates to humans, we investigated SATB1 functions in regulation of Wnt/Wg signaling by using mammalian cell-lines and *Drosophila*. Here, we present evidence that in mammalian cells, SATB1 interacts with Dishevelled, an upstream component of the Wnt/Wg pathway. Conversely, ectopic expression of full-length human SATB1 but not that of its N- or C-terminal domains in the eye imaginal discs and salivary glands of third instar *Drosophila* larvae increased the expression of Wnt/Wg pathway antagonists and suppressed phenotypes associated with activated Wnt/Wg pathway. These data argue that ectopically-provided SATB1 presumably modulates Wnt/Wg signaling by acting as negative regulator in *Drosophila*. Interestingly, comparison of SATB1 with PDZ- and homeo-domain containing *Drosophila* protein Defective Proventriculus suggests that both proteins exhibit limited functional similarity in the regulation of Wnt/Wg signaling in *Drosophila*. Collectively, these findings indicate that regulation of Wnt/Wg pathway by SATB1 is context-dependent.

## Introduction

SATB1 is a genome organizer involved in the regulation of multiple genes. Functionally characterized domains of SATB1 include the N-terminal PDZ-like domain and a C-terminal DNA-binding region comprising a CUT domain and a homeodomain^1,2^. The N-terminal of SATB1 acts as the principal interface for its dimerization as well as protein interactions^3,4^. The C-terminal half of SATB1 is required for DNA-binding, which is dependent upon dimerization mediated by the N-terminal domain^5^. Most proteins harboring a PDZ domain are localized in the cytosol and are often involved in the organization of multiprotein complexes^6,7^. In contrast, SATB1 is one of the few known nuclear proteins that harbor a PDZ-like domain^8^ and possibly functions as a scaffold for anchoring various proteins to their target genomic loci^4,9^. SATB1 also features a nuclear matrix targeting sequence (NMTS) spanning residues 224 to 278 that is required for the binding of SATB1 to the nuclear matrix^10^. The N-terminal region of SATB1 harboring the PDZ-like domain has recently been shown to fold as a ubiquitin-like domain (ULD) based on the 3-D structure determined by X-ray crystallography^11^. ULD is also known to be involved in mediating protein–protein interactions^12^. The role of SATB1 as a regulator of transcription has been well studied^13–15^ however, the signaling mechanisms that regulate SATB1 functions during transcription remain to be elucidated. Understanding the interaction of SATB1 with components of different signaling pathways will provide important insights about its function under specific developmental contexts.

Alterations in SATB1 protein levels result in deregulation of various genes at a global level. Many of these genes are involved in signaling pathways such as those mediated by TGF-β and Wnt/Wg^16^. Previous studies using human thymocytes have shown that SATB1 physically interacts with β-catenin and also regulates a common set of genes during thymocyte development^3^. These results suggest a possible cross-talk between SATB1 and the components of the Wnt/Wg signaling pathway. Studies have also suggested that the ectopically-expressed N-terminal region of SATB1 acts as a dominant negative regulator of SATB1 function in HEK293 cell line and thymocytes^16^. Therefore, to further understand the role of SATB1 in regulation of Wnt/Wg signaling, we tested the interaction between the N-terminal domain of SATB1 and Dvl another PDZ-containing component of the Wnt pathway. We further studied the effects of ectopic expression of SATB1 on Wnt/Wg signaling by employing both mammalian cell-line system and *Drosophila* as model systems.

## Results

### SATB1 interacts with the Wnt pathway component Dishevelled (Dvl) and may function as part of the same complex

**Figure 1:**
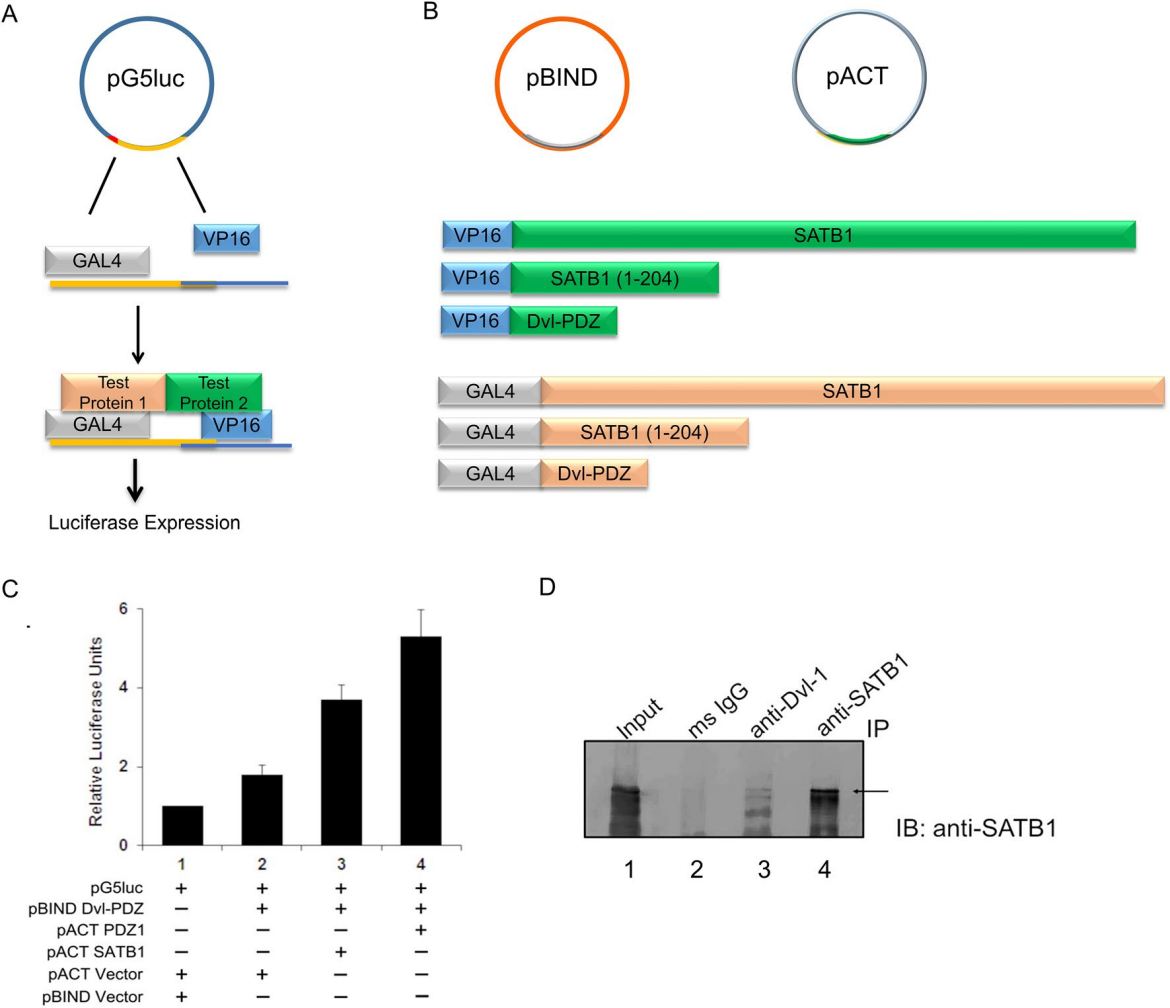
SATB1 physically and functionally interacts with Dishevelled-1 (Dvl) in cell lines. (**A**) Schematic representation of the CheckMate mammalian two-hybrid system. The pG5luc Vector contains GAL4 binding sites (orange) upstream of a minimal TATA box (red), which in turn is upstream of the firefly luciferase gene. The pBIND vector contains GAL4 and the pACT contains the VP16 activation domain. Interaction between the test proteins, expressed as GAL4 and VP16 fusion constructs, results in luciferase expression. (**B**) Schematic representation of the constructs used in the mammalian two-hybrid assay**.** The GAL4 in the pBIND vector and VP16 activation domain of the pACT vector have been shown in grey and green, respectively. The GAL4 and VP16 fusion constructs have also been shown using the same color coding used for the test proteins in A. Note that SATB1 (1-204) has been denoted as PDZ1 in C. (**C**) Mammalian two-hybrid assay. Four-fold increase in reporter activity was observed upon co-transfection of Dvl-PDZ along with full-length SATB1 as compared to control (bar 3 versus bar 1). Co-transfection of Dvl-PDZ and SATB1 (1-204) (PDZ1) resulted in fivefold increase in reporter activity with respect to control (bar 4 versus bar 1). (**D**) Co-immunoprecipitation assay. SATB1 was overexpressed in HEK293 cells and co-immunoprecipitation was performed as described in ‘Methods’. Immunoprecipitation using Dvl-1 antibody followed by immunoblot using SATB1 antibody resulted in a band corresponding to the apparent molecular weight of SATB1 at 105 kDa (lane 3). Immunoprecipitation using SATB1 antibody also yielded a band of the same size (lane 4, depicted by an arrow).

Since SATB1 is known to mediate Wnt signaling by interaction with β-catenin, we investigated whether SATB1 exerts its effect via physical interaction with any other components of the Wnt pathway. Based on in silico domain architecture analysis, we identified Dishevelled (Dvl) among the Wnt pathway components as a potential interacting protein of SATB1. Both these proteins comprise a PDZ-like domain which is known to be an interface for protein interactions. To investigate PDZ-like domain-mediated interactions between SATB1 and Dvl-1, mammalian two-hybrid assay (M2H) was employed^17,18^. Full length SATB1 or the N-terminal region harboring the PDZ-like domain of SATB1 were cloned in the pACT vector. HEK293 cells were co-transfected with pBIND-DVL-PDZ fusion construct and the pACT-SATB1 (1-204) or pACT full-length SATB1 constructs using pG5luc as the reporter vector. Mammalian two-hybrid assay was used to investigate the interaction between Dishevelled and SATB1 (Fig. 1A,B). This assay suggested that SATB1 and Dishevelled (Dvl/Dsh) might be the part of the same complex. Cotransfection of HEK293 cells with pBIND-Dvl-PDZ and either pACT-SATB1 (1-204) or pACT-SATB1 yielded fourfold and 2.5-fold increase in the reporter activity compared to the control, respectively (Fig. 1C). These results suggest that the PDZ-like domain of human SATB1 is required for complex formation with Dishevelled (Dvl-1). For confirming whether SATB1 and Dishevelled (Dvl-1) physically interact in vivo, co-immunoprecipitation assay was performed. Anti-Dvl-1 antibody was used to immunoprecipitate Dishevelled-1, followed by immunoblotting with anti-SATB1 antibody to monitor the association of SATB1 with Dishevelled-1. We observed that anti-Dvl antibody immunoprecipitated SATB1, indicating that Dvl-1 and SATB1 exist as part of a protein complex in vivo (Fig. 1D, Figure S1).

### SATB1 positively regulates components of the Wnt/Wg pathway and Wnt-responsive genes

**Figure 2:**
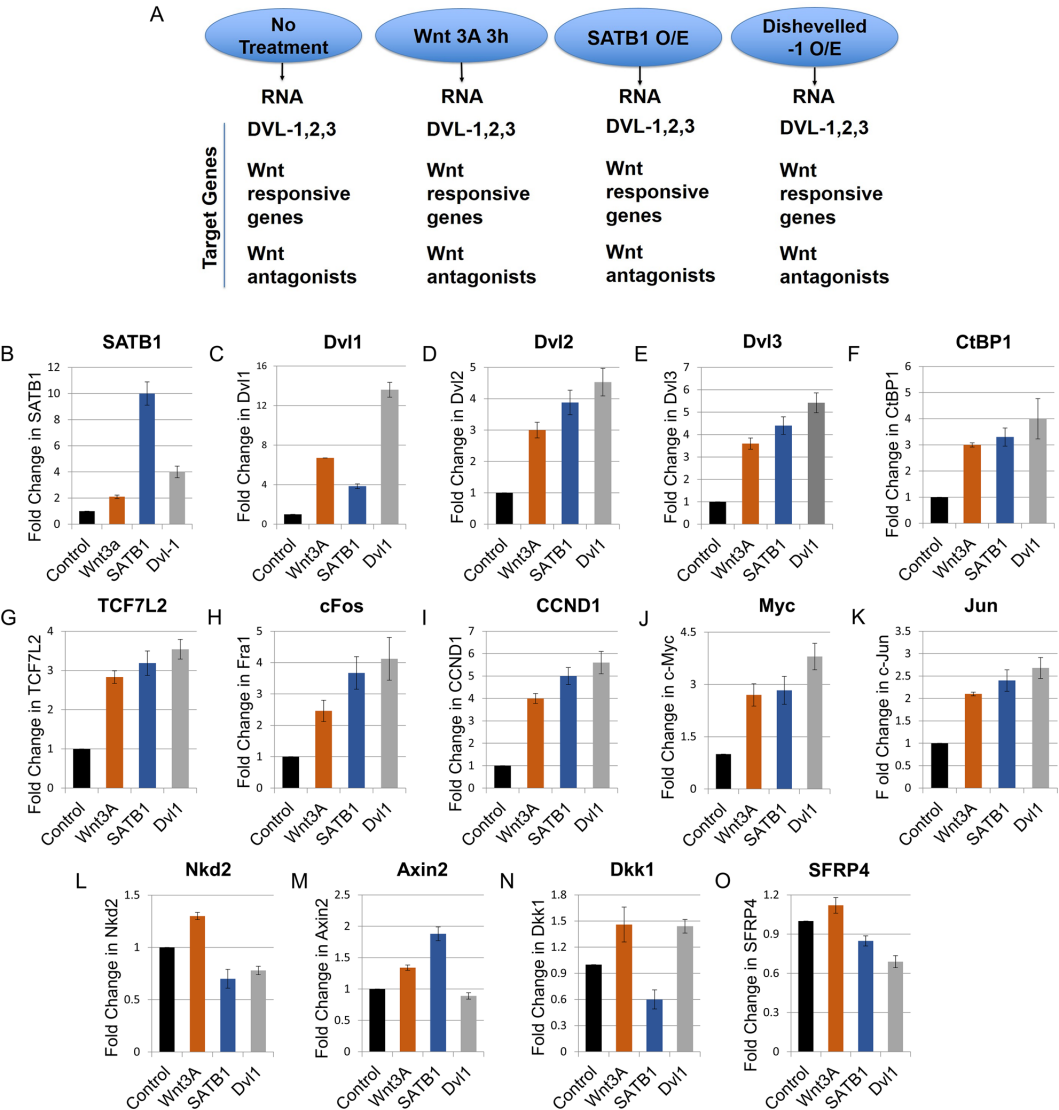
SATB1 is a positive regulator of the Wnt/Wg pathway. (**A**) Flow-chart representation of sample preparation for quantitative RT-PCRs. (**B**) Quantitative RT-PCR revealed a fourfold increase in SATB1 transcript upon overexpression of Dishevelled-1 (N = 3). (**C**) A 3.8-fold increase in Dvl-1 transcript was observed upon SATB1 over-expression. (**D**,**E**) SATB1 overexpression resulted in upregulation of the transcript levels of Dvl2 and Dvl3 homologs of Dishevelled. (**F**) levels of CtBP1 transcript upon overexpression of SATB1 or Dvl or Wnt3A treatment. (**G**) levels of TCF7L2 transcript upon overexpression of SATB1 or Dvl or Wnt3A treatment. (**H**) cFOS/Fra-1 transcript levels upon overexpression of SATB1 or Dvl or Wnt3A treatment. (**I**) cyclinD1 (CCND1) transcript levels upon overexpression of SATB1 or Dvl or Wnt3A treatment. (**J**) c-myc transcript levels upon overexpression of SATB1 or Dvl or Wnt3A treatment (**K**) c-Jun transcripts upon overexpression of SATB1 or Dvl or Wnt3A treatment. (**L**) Nkd2 transcript upon overexpression of SATB1 or Dvl or Wnt3A treatment. (**M**) Axin2 transcript levels upon overexpression of SATB1 or Dvl or Wnt3A treatment. (**N**) Dkk transcripts upon overexpression of SATB1 or Dvl or Wnt3A treatment. (**O**) SFRP4 transcripts upon overexpression of SATB1 or Dvl or Wnt3A treatment. All RNAs were treated with DNase prior to conversion into cDNA. 18S RNA was used as an internal control for normalizing the CT values. Fold-change was calculated as described in the ‘Methods’. Error bars indicate standard deviation calculated from triplicates (N = 3). Fold-change as compared to control is depicted on Y-axis.

SATB1 can form a complex involving Dishevelled (this study) and β-catenin^3^. To study the role of SATB1 in regulation of Wnt/Wg signaling in the mammalian system, the transcript levels of various Wnt/Wg components were measured by quantitative RT-PCR after the following treatments: a) treatment with Wnt3A (3 h); b) overexpression of SATB1; c) overexpression of Dishevelled-1 in HEK293 cells (scheme depicted in Fig. 2A. Quantitative RT-PCR analysis revealed a fourfold increase in SATB1 transcript upon overexpression of Dishevelled-1 and a concomitant 3.8-fold increase in Dvl-1 transcript upon SATB1 overexpression (Fig. 2B, bar 4 and Fig. 2C bar 3, respectively). Since overexpression of Dvl or Wnt3A treatment led to the upregulation of SATB1 expression, it can be inferred that SATB1 is regulated via the Wnt/Wg pathway (Fig. 2B). SATB1 overexpression also upregulated the transcript levels of Dvl2 and Dvl3 homolog of Dishevelled (bar 3, Fig. 2D,E, respectively).

Further, SATB1 overexpression in HEK293 resulted in an increase in the transcript levels of Wnt-responsive transcriptional factors such as CtBP and TCF7L2 (Fig. 2F,G, bar 3). The transcript levels of other Wnt-responsive genes such as c-FOS (Fra-1), cyclinD1 (CCND1), c-myc, and c-Jun also increased in response to the overexpression of SATB1 (Fig. 2H,I,J,K, bar 3). These Wnt-responsive genes were similarly upregulated upon overexpression of Dishevelled-1 or Wnt3A treatment (Figures, 2H, 2I, 2J and 2K, bars 4 and bar 2, respectively) with the exception of Axin2 which remained unchanged (Fig. 2M). Further, SATB1 overexpression also led to the repression of Wnt/Wg pathway antagonists, Nkd2, Dkk1, and SFRP4 (Fig. 2L,N,O, bar 3). Similar result was observed in case of Dvl-1 overexpression for Nkd2 and SFRP4 (Fig. 2L,O, respectively bar 4), but Dkk1 did not follow the same trend (Fig. 2N). Treatment with Wnt3A did not cause much change in the transcript levels of Nkd2, Dkk1, and SFRP4 (Fig. 2L,N,O, bar 2). This was expected since these genes are known to be expressed at later time points in response to Wnt3A stimulation thereby leading to feed-back inhibition of Wnt/Wg signaling pathway. Our results therefore establish a positive correlation between SATB1 and activation of the Wnt/Wg pathway.

### Ectopically-expressed SATB1 suppresses development defects resulting from overexpression of Wnt/Wg components in eye and wing imaginal discs of Drosophila larvae

To study the correlation between SATB1 and Wnt/Wg signaling further in an in vivo system*,* we decided to employ *Drosophila melanogaster* as a model system for two reasons, i) There is no obvious SATB1 homolog in the Drosophila genome and, as a result there is no endogenous source of SATB1 protein, and ii) by contrast, it expresses most of the canonical components of the Wnt/Wg pathway needed for the regulation of Wnt/Wg signaling.

The adult *Drosophila* eye is a compound structure comprised of 750 ommatidia that develop during the third larval instar stage as the morphogenetic furrow progresses from posterior to anterior in the eye disc^19^. The *GMR* promoter is expressed posterior to the morphogenetic furrow in developing photoreceptor cells^20^. Wingless signaling pathway is known to be involved in the fate determination of cells in the eye and those in head cuticle^21–23^. Misexpression of Wnt components using *GMR-Gal4* driver disrupts photoreceptor and ommatidial bristle formation and therefore this system provides a suitable model system to study regulation of Wnt/Wg signaling in vivo. As described earlier, SATB1 was shown to associate with Dvl in mammalian cell lines, the interaction between SATB1 and the *Drosophila* homolog of Dvl (Dsh) was investigated in the developing *Drosophila* eye.

**Figure 3:**
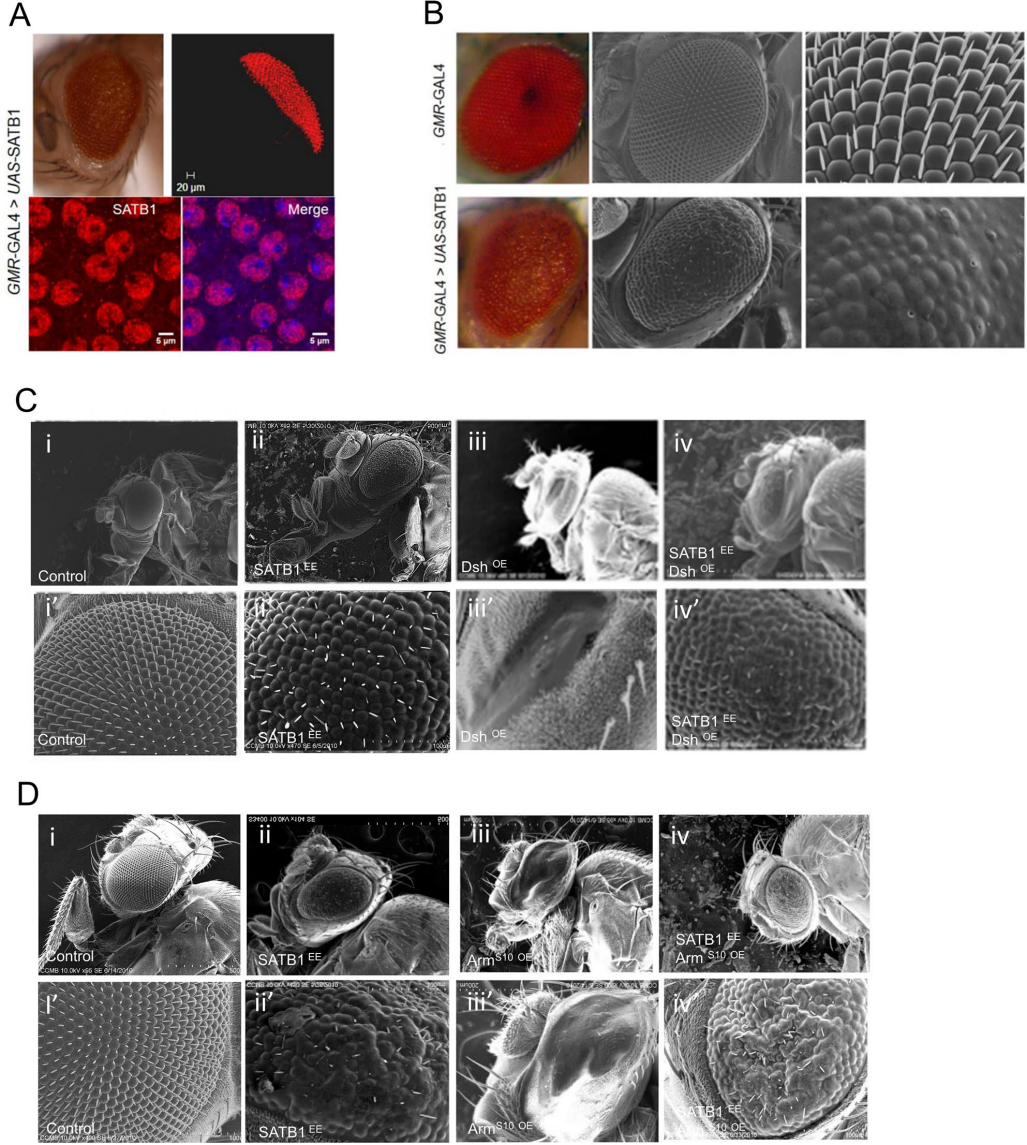
Subcellular localization of SATB1 is comparable in mammalian cell lines and cells of the eye imaginal discs. (**A**) Ectopic expression of human SATB1 in the fly eye gives rise to rough eye. Immunostaining for ectopically-expressed SATB1 (red) and DNA (blue) in the eye imaginal disc revealed that SATB1 is localized in the region posterior to the morphogenetic furrow (top panel). Higher magnification immunofluorescence images reveal that similar to that in the mammalian system, SATB1 localizes entirely within the nucleus, while completely excluding the heterochromatic regions. (**B**) Scanning electron microscopic (SEM) images depicting the fusion of ommatidia as a result of ectopic expression of full-length SATB1. SEM imaging of *GMR-Gal4* > UAS-*SATB1* fly eyes revealed that ommatidia have lost their wild type hexagonal shape and the compact arrangement. Inter-ommatidial bristles are randomly arranged while some regions of the eye completely lack bristles (Penetrance 100%, n = 110). (**C**) Small-eye phenotype due to Dsh overexpression is suppressed by expression of human SATB1. (**i**) Control *GMR-GAL4*/+. (**ii**) Fly eyes ectopically expressing SATB1. (**iii**) Fly eyes ectopically expressing Dsh have small eyes. (**iv**) Fly eyes expressing SATB1 in the background of Dsh misexpression have large eyes as compared to *GMR*-Gal4 > UAS-*dsh* (Penetrance of this phenotype is 30%, n = 200) (**D**) Small-eye phenotype due to expression of activated arm^S10^ is suppressed by expression of human SATB1. (**i**) Control *GMR-GAL4*/+. (**ii**) Fly eyes ectopically expressing SATB1. (**iii**) Fly eyes ectopically expressing ArmS10 exhibit the small-eye phenotype. (**iv**) Flies expressing SATB1 in the background of ArmS10 expression have large eyes as compared to *GMR*-Gal4 > UAS-*arm*^*S10*^ (Penetrance of this phenotype is 20%, n = 174) (I”), (ii’), (iii’), and (iv’) in C and D are higher magnification images. At all stages *UAS-GFP* was used as control.

Ectopic expression of human SATB1 induced under the control of *GMR-GAL4* driver resulted in reduction in the size of the adult eyes as well as a rough eye phenotype due to misalignment of ommatidia. Immunostaining for monitoring the localization of SATB1 in the eye imaginal disc revealed that SATB1 expression is restricted to the posterior part of the eye imaginal disc (Fig. 3A, top panel). The pattern of SATB1 expression and localization in the eye imaginal disc correlated with the expression domain of *GMR-GAL4.* Similar to the localization of SATB1 in mammalian thymocytes, ectopically-expressed SATB1 localized entirely inside the nucleus and was observed to be enriched in DAPI-poor regions and excluded from DAPI-rich heterochromatic regions (Fig. 3A, bottom panel). Analysis of adult eyes by scanning electron microscopy (SEM) revealed that ectopic expression of SATB1 severely distorted the eye morphology due to fusion of ommatidia and disruption of bristle organization (Fig. 3B, compare lower panel with upper panel). These phenotypes were evenly spread throughout the eye and were not restricted to any particular region. Elevated *dsh* level in the eye is known to result in ommatidial degeneration and cell death leading to severe reduction in the size of the eye^24^. SEM analysis of transgenic *Drosophila* expressing Dsh under the control of *GMR-GAL4* revealed a drastic reduction in size, complete fusion of ommatidia, absence of bristles leading to a glossy eye phenotype (Fig. 3C, iii and iii’).

Ectopic expression of SATB1 simultaneously with overexpression of *Dsh* resulted in a modest suppression of the eye phenotype observed due to overexpression of only *Dsh*. These results suggest that ectopic expression of SATB1 in the eye imaginal disc in *Drosophila* is sufficient to suppress the phenotypes caused by overexpression of Dsh, contrary to its role in activation of the Wnt/Wg pathway as observed in the mammalian system. These studies further indicate that the interaction between SATB1 and Wnt components such as Dishevelled is context dependent (Fig. 3C, iv and iv’).

Commitment to the Wnt/Wg pathway is fully established with the stabilization and translocation of β-catenin or Armadillo to the nucleus, where it leads to the activation of Wnt/Wg-responsive genes. We analyzed the effect of SATB1 ectopic expression in the background of constitutive expression of *arm* (Arm^S10^). Arm^S10^ is a mutant which is constitutively activated due to stabilization of the ARM protein. Ectopic expression of activated *Arm*^*S10*^ mimics constitutive activation of the Wingless signaling cascade^25,26^. Experiments performed in thymocytes and cell-lines revealed that SATB1 physically interacts with β-Catenin^3^. Overexpression of constitutively-activated ARM using the eye-specific *GMR-GAL4* driver resulted in drastic reduction of the eye size. SEM analysis revealed completely fused ommatidia along with bristle loss similar to that observed upon Dsh overexpression (Fig. 3D, iii and iii’). Simultaneous expression of SATB1 and activated ARM resulted in larger eyes as compared to those observed in flies expressing constitutively-activated ARM protein. However, these flies also exhibited rough eye phenotype (Fig. 3D, iv and iv’). Transgenic flies expressing *UAS-GFP* under the control of *GMR–GAL4* were used as controls*.* This result is in agreement with the hypothesis that ectopic expression of human SATB1 might regulate the components of Wnt/Wg pathway in *Drosophila*.

### Ectopically-expressed SATB1 N-terminal (1-204) region fails to suppress phenotypes associated with overexpression of Wnt/Wg components

**Figure 4:**
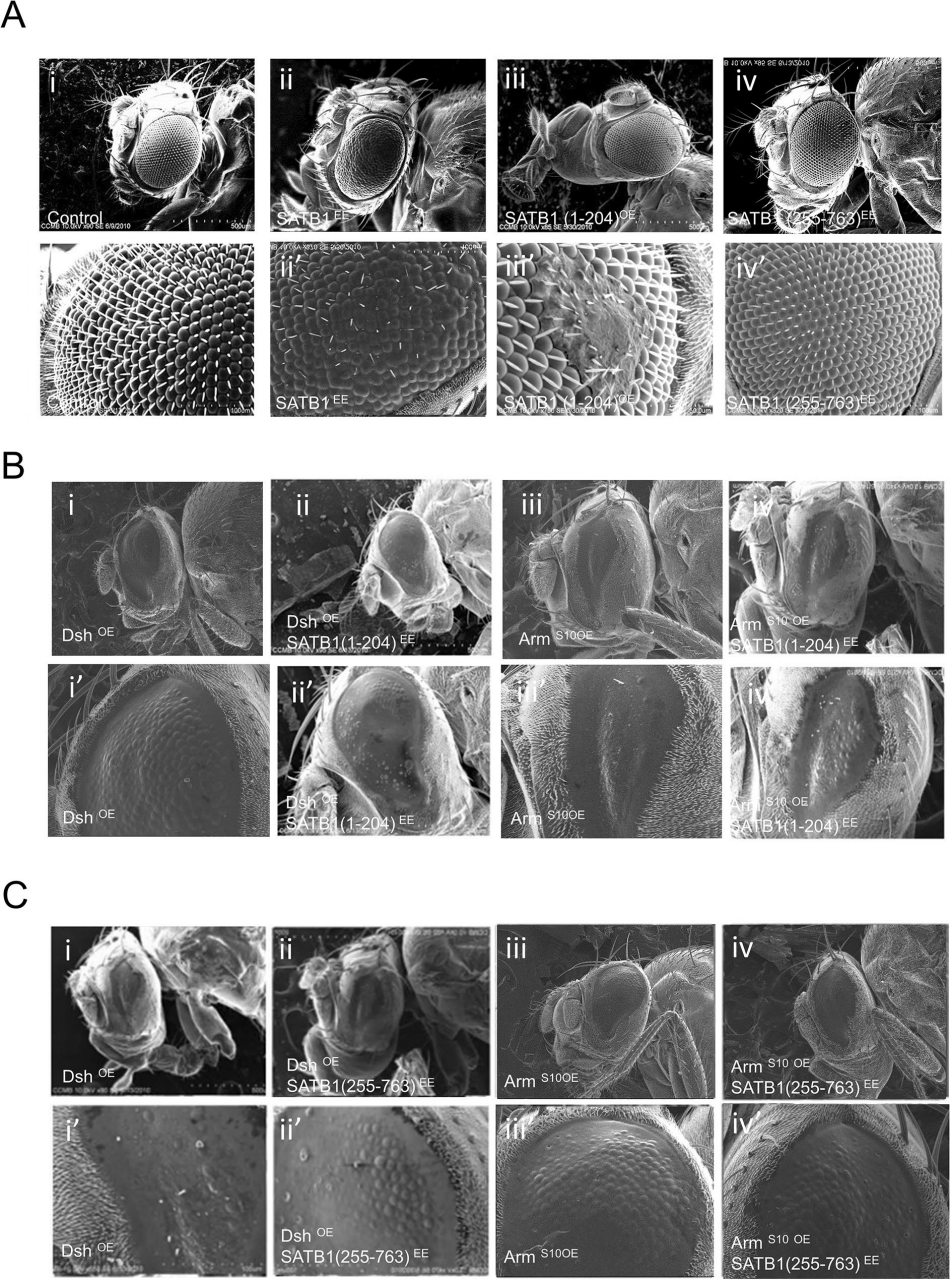
Ectopic expression of SATB1 N-terminal (1-204) region fails to suppress phenotypes associated with overexpression of Wnt/Wg components. (**A**) SEM images of fly eyes expressing SATB1 and SATB1 deletion constructs under the regulation of *GMR-*GAL4. (**i**) Control, *GMR-GAL4*/+. (**ii**) Eyes ectopically expressing SATB1. (**iii**) Eyes ectopically expressing SATB1 (1-204). (**iv**) Eyes ectopically expressing the (255-763) region of SATB1. (**B**) The N-terminal PDZ-like domain does not suppress the small-eye phenotype of Dsh misexpression. (**i**) Fly eyes ectopically expressing Dsh. (**ii**) Fly eyes expressing SATB1 (1-204) in the background of Dsh overexpression. (**iii**) Fly eyes ectopically expressing ArmS10. (**iv**) Fly eyes expressing SATB1 (1-204) in the background of ArmS10 overexpression. **C,** Ectopic expression of the DNA-binding domain of SATB1 does not suppress the small-eye phenotype generated upon misexpression of Dsh. (**i**) Fly eyes ectopically expressing Dsh. (**ii**) Fly eyes expressing SATB1 (255-763) in the background of Dsh overexpression. (**iii**) Fly eyes ectopically expressing ArmS10. (**iv**) Fly eyes ectopically expressing SATB1 (255-763) in the background of ArmS10 overexpression. At all stages, *UAS-GFP* was used as control. (**I**”), (**ii**’), (**iii**’)**,** and (**iv’**) in B and C are higher magnification images.

To identify the specific domain that is sufficient to produce a rough eye phenotype in *Drosophila*, various functional domains of SATB1 were expressed in the *Drosophila* eye using *GMR-GAL4*. The N-terminal (1-204) domain is required for interaction with other proteins and SATB1 homodimerization whereas the C-terminal (255-763) region is involved in DNA-binding^4^. Ectopic expression of SATB1-(1-204) in the developing eye resulted in loss of ommatidial structure and bristle arrangement in some regions in the eye but no change in the size of the eye was observed as revealed by SEM analysis (Fig. 4A, iii and iii’; 70% penetrance). In contrast, *GMR-GAL4-*driven expression of the DNA-binding region of SATB1 (255-763) in the developing eyes did not result in any morphologically discernible phenotypes as revealed by SEM analysis. Flies with the *UAS-SATB1 (255-763)/GMR-GAL4* genotype exhibited compactly packed and hexagonal arrangement of ommatidia separated by uniformly spaced bristle as observed in wildtype (Fig. 4A, iv and iv’). This is in concordance with our hypothesis that in the absence of the N-terminal domain-mediated dimerization, SATB1-(255-763) does not bind to DNA and hence might not manifest in a phenotype. However, it might also be possible that SATB1-(1-204) and SATB1-(255-763) proteins might not be as stable as full length SATB1, and that this might have resulted in the observed phenotypes or the lack thereof. According to the cell line data, SATB1 physically and functionally interacts with Dishevelled via its N-terminal PDZ-like domain, the expression of which acts as dominant negative regulator for SATB1 function^16^. Ectopic expression of specific SATB1 domains as described earlier was induced in *UAS-dsh/GMR-GAL4* backgrounds to investigate whether the expression of any of these domains would be sufficient to suppress the small eye phenotype caused by overexpression of *dsh. UAS-SATB1/GMR-GAL4; UAS-dsh* flies exhibited completely fused ommatidia and smaller eyes, similar to that observed in *Drosophila* expressing *UAS-dsh* under the control of *GMR-GAL4* with a penetrance of 100% (Fig. 4B, ii and ii’ versus i and i’). This suggests that the ectopic expression of only the N-terminal of SATB1 (1-204) was not sufficient to suppress the phenotype observed due to overexpression of *dsh.* SEM analysis of transgenic flies expressing *UAS-SATB1 (255*-*763)/GMR-GAL4; UAS-dsh* revealed that the expression of only the DNA-binding domain of SATB1 did not suppress the small eye phenotypes associated with overexpression of *dsh* using *UAS-dsh/GMR-GAL4* flies (Fig. 4C, ii and ii’ versus i and i’). These results indicate that interaction between *Drosophila* DSH and SATB1 requires both the N-terminal PDZ-containing domain and the C-terminal DNA-binding domain of SATB1.

The N-terminal protein interaction domain of SATB1 has been shown to physically interact with the C-terminal transactivation domain of β-catenin^3^. Further, to analyze the effects of ectopic expression of the SATB1 (1-204) domain in cells where the Wnt/Wg is constitutively active, SATB1 (1-204) was expressed simultaneously with constitutively-activated ARM protein using *GMR-GAL4* (Fig. 4B, ii, ii’). Ectopic expression of N-terminal domain of SATB1 (1-204) in *UAS-SATB1 (1*-*204)/GMR-GAL4; UAS-arm*^*S10*^ genotype *Drosophila* was not sufficient to suppress the small eye phenotypes associated with constitutive ARM^S10^ expression in the eye (Fig. 4B, iii and iii’ versus i and i’). Similarly, ectopic expression of the DNA-binding domain of SATB1 (255-763) simultaneously with the expression of constitutively active Arm^S10^ using *GMR-GAL4* did not suppress the small eye phenotype (Fig. 4C, iv and iv’ versus i and i’). These results suggest that similar to DSH, the interaction of ARM with SATB1 is also mediated via both the N-terminal protein-binding and the DNA-binding domains.

### Ectopic expression of SATB1 affects expression of Wnt responsive genes in the Drosophila eye disc

**Figure 5:**
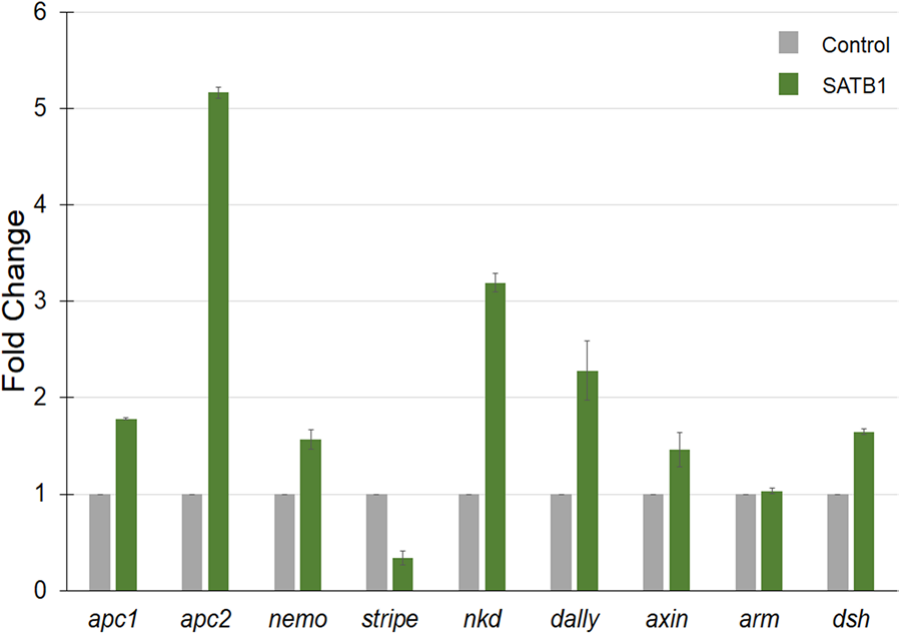
SATB1 upregulates the antagonists of the Wnt pathway. Total RNA was isolated from the eye imaginal discs upon ectopic expression of SATB1 as described in ‘Methods’. Quantitative RT-PCRs were performed to monitor the expression levels of genes involved in the Wnt/Wg signaling pathway in the eye imaginal discs of control larvae (Grey bars) and larvae expressing SATB1 using GMR-Gal4 (green bars). Rp49 was used as an internal control. Error bars indicate standard deviation calculated from triplicates (N = 3). Fold change as compared to control is depicted on Y-axis.

Our study revealed that SATB1 associates with the Wnt component Dishevelled and activates Wnt pathway in the mammalian system whereas in *Drosophila*, ectopic expression of SATB1 is sufficient to suppress the phenotypes due to over-expression of *Dsh.* To better understand the mechanism underlying the regulation of Wnt/Wg pathway by SATB1-like molecules in different developmental contexts we analyzed the transcript levels of Wnt responsive genes and Wnt antagonists upon ectopic expression of SATB1 in the eye imaginal discs by qRT-PCR. SATB1 was expressed using *GMR-GAL4*. The transcript levels of Wnt-responsive genes such as *dsh*, *stripe* (*sr*), *axin*, *nemo*, *nkd*, *apc1*, and *apc2* were quantified in the eye disc. Of these, *dally*, *axin*, *nkd*, *apc1*, and *apc2* are known to antagonize the Wnt/Wg pathway, *stripe* and *nemo* are Wnt targets, and *dsh* expression correlates with Wnt activation^6,7,27–30^. Upon SATB1 expression, the transcript levels of genes which antagonize the Wnt/Wg pathway such as *dally*, *apc-2*, and *nkd* were increased approximately twofold, fivefold and threefold, respectively, whereas a 0.6-fold decrease was observed in the transcript levels of Wnt-responsive genes such as *stripe* (Fig. 5). While over-expression of SATB1 in the mammalian cells resulted in a significant tenfold increase in *dsh* transcript levels; ectopic expression of SATB1 in the *Drosophila* eye led to a modest increase of 1.5-fold in the transcript level of *dsh* (Fig. 5)*.* Taken together, the increased transcript levels of Wnt/Wg pathway antagonists such as *apc2*, *nkd* and *dally*, along with very mild increase in *dsh* transcript might be responsible for the suppression of Wnt/Wg pathway and phenotypes associated with Wnt/Wg activation upon ectopic expression of SATB1.

To elucidate the mechanism underlying the upregulation of Wnt/Wg pathway antagonists *nkd* and *apc-2* upon ectopic expression of SATB1, the regions upstream to the transcription start sites of these genes were analyzed for the presence of consensus SATB1 binding sites^4^. Motif search analysis was performed using the MEME suite. Putative SATB1 binding sites were observed in the upstream regions of both *nkd* and *apc-2*, suggesting that ectopically expressed SATB1 can potentially bind to the regulatory regions of these genes (Supplementary Figure S2).

### Human SATB1 can bind to Drosophila chromatin

**Figure 6:**
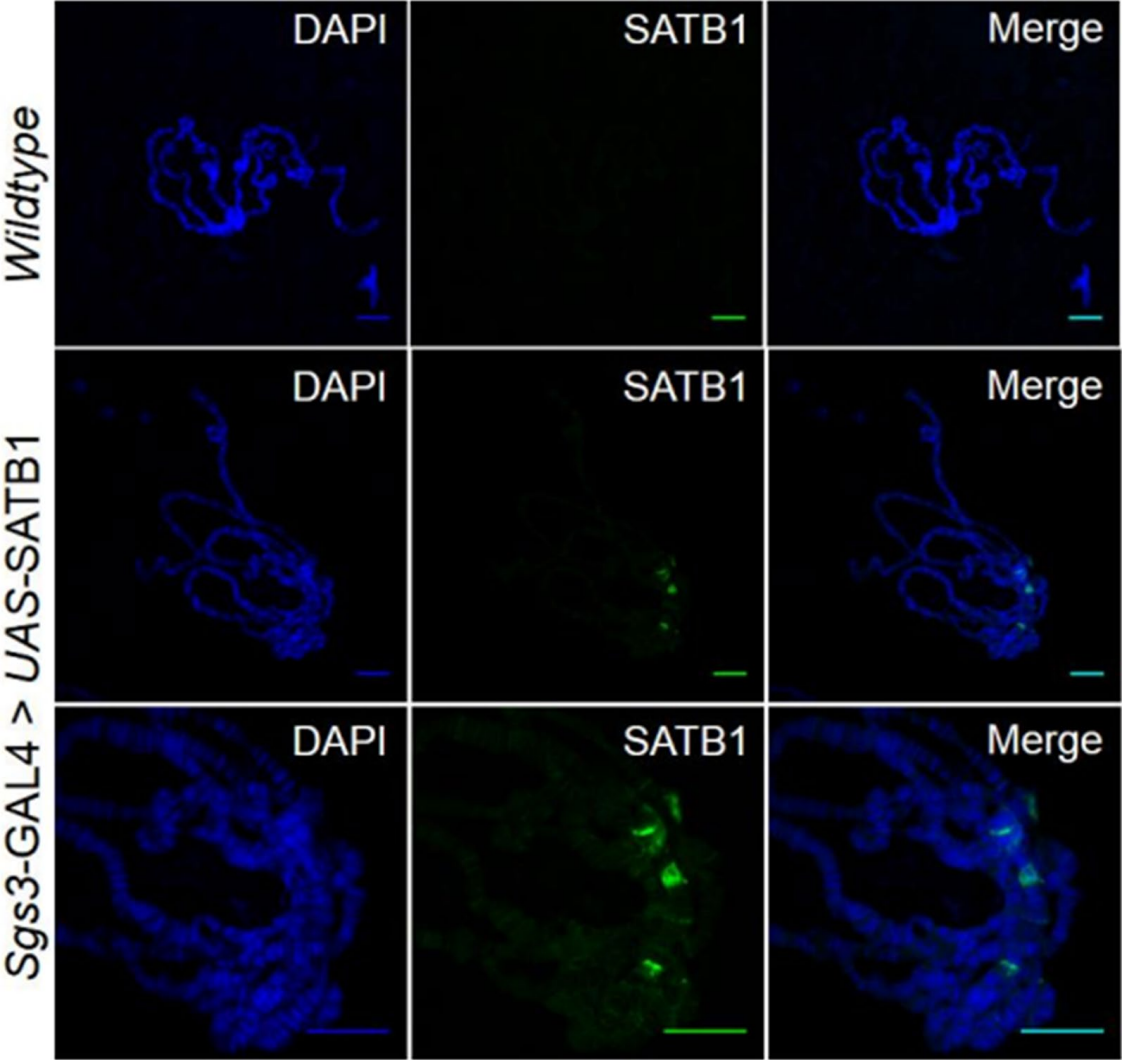
Mammalian SATB1 can bind Drosophila chromatin and regulate expression of genes. SATB1 binds *Drosophila* chromatin. SATB1 was ectopically expressed in the salivary glands of third instar larvae as described in the ‘Methods’. Bands corresponding to SATB1 (green) were observed in case of *Sgs3-GAL4* > UAS-*SATB1* but were totally absent in control spreads. Images were acquired at 40 × magnification. Lower panel depicts the staining for SATB1 at 100 × magnification. All the scale bars represent 20 μm.

The phenotypes observed due to ectopic expression of human SATB1 in the *Drosophila* eye could result from SATB1 binding to *Drosophila* chromatin or sequestration of endogenous proteins by SATB1. To distinguish between the two possibilities, binding of SATB1 to the *Drosophila* chromatin was assayed by immunostaining of salivary gland polytene chromosome spreads using an antibody against human SATB1 in *UAS-SATB1/Sgs3-GAL4* genotype flies. We observed specific SATB1 staining on the *Drosophila* chromatin, which was particularly intense in four regions present in the vicinity of the chromocenter (Fig. 6). The *Drosophila* boundary element associated MAR-binding protein BEAF was used as a positive control for staining. Bands corresponding to characteristic pattern of BEAF binding were observed, confirming that the staining method was optimal (Supplementary Figure S3). Additionally, multiple bands with lower intensity were interspersed throughout the extended arms of chromosomes suggesting the occurrence of multiple SATB1 binding sites throughout the fly genome. In concordance with this, 21 putative SATB1-binding sites were identified in the *Drosophila* genome by employing motif finding analysis using MEME (Supplementary Figures S2 and S4) that used the consensus SATB1-binding site (CSBS) as query sequence^4,9^. These loci were represented on both chromosome II and chromosome III, suggesting that multiple putative SATB1-binding sites exist in the fly genome. These results indicate that mammalian SATB1 can potentially bind *Drosophila* chromatin in vivo*.*

### PDZ domain-containing Defective Proventriculus (DVE) regulates Wnt/Wg components in Drosophila eye

Earlier studies from our laboratory have established that the interaction between SATB1 and β-catenin is mediated via the N-terminal PDZ-like domain of SATB1^3^. Since SATB1 is absent in *Drosophila*, we decided to identify similar PDZ domain-containing proteins in *Drosophila* that play a crucial role in regulation of Wnt/Wg signaling and other pathways.

**Figure 7:**
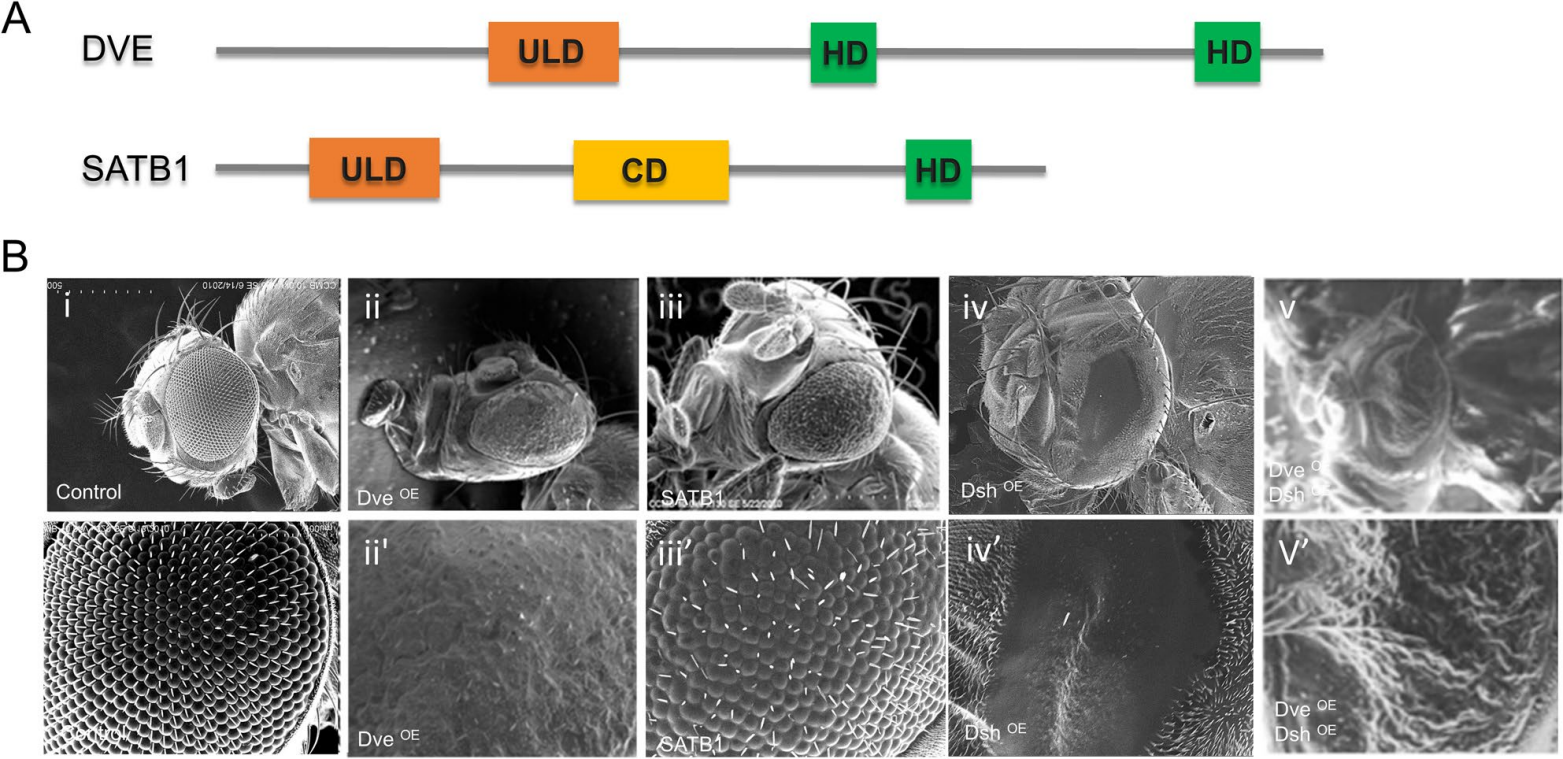
Domain organization of *Drosophila* Defective proventriculus protein (DVE) in comparison to SATB1. (**A**) Domain architecture of SATB1 and DVE. SATB1 harbors a PDZ-like domain region spanning amino acids (90-204), this region is also characterized as COMPASS domain and has recently been shown to fold like ubiquitin-like domain (ULD, orange box). The C-terminal region of SATB1 spanning amino acids 2555-763 harbors a CUT domain (CD, yellow box) and a homeodomain (HD, green box). Defective proventriculus (DVE) harbors a conserved PDZ-like N-terminal domain, which exhibits a high degree of similarity with the PDZ-like domain of SATB1 that is shown to fold like the ULD (orange box). In its C-terminal DVE harbors two homeodomains (green boxes), though they exhibit limited similarity with SATB1 at the level of amino acid residues. (**B**) Ectopic expression of *dve* gives rise to a rough eye phenotype similar to SATB1, and DVE suppresses the small-eye phenotype due to Dsh overexpression. (**i**) Control *GMR-GAL4*/+. (**ii**) Fly eyes ectopically expressing DVE (Penetrance of this phenotype is 100, n = 146) (**iii**) Fly eyes ectopically expressing mammalian SATB1. (**iv**) Fly eyes ectopically expressing Dsh. (**v**) Fly eyes expressing DVE in the background of Dsh overexpression. At all stages *UAS-GFP* was used as control. (**i’**), (**ii’**), (**iii’**), (**iv’**), and (**v’**) are higher magnification images of the same.

Domain structure analysis of COMPASS family of proteins revealed that the *Drosophila* protein Defective Proventriculus (DVE) is most similar to vertebrate SATB1^2^. The PDZ-like domains of these proteins share 62% amino acid identity (also predicted to be a COMPASS domain)^25,31^. In contrast, the C-terminal of DVE contains two homeodomains unlike SATB1 that comprises a CUT domain and a homeodomain (Fig. 7A). However, the homeodomains in DVE and SATB1 do not display significant similarity^2^. In *Drosophila*, DVE is usually expressed in the proventriculus, midgut, the leg, wing, eye, and antennal imaginal discs, during the larval stages, and the midgut during adult stages. Since DVE has been shown to suppress Wnt/Wg signaling during the development of proventriculus, eye and wing, we decided to assess the correlation between DVE and SATB1 expression and Wnt/Wg signaling in these developmental contexts^32–35^. Ectopic expression of *dve* in the developing eye of the *Drosophila* using a *GMR-GAL4*, resulted in fusion of ommatidia and bristle loss and a rough eye phenotype. Based on SEM analysis, the phenotype observed in *GMR-GAL4; UAS-dve* was far more severe compared to that observed in *UAS-SATB1/GMR-GAL4* flies (Fig. 7B, compare panels ii and ii’ with iii and iii’). To assess whether as in the case of SATB1, overexpression of DVE is sufficient to suppress the gain of function phenotypes of *dsh, dve* was simultaneously overexpressed with *dsh* in the developing eye using *GMR-GAL4*. SEM analysis of *UAS-dsh/GMR-GAL4* genotype *Drosophila* revealed complete fusion of ommatidia and total absence of bristles and a drastic reduction in size (Fig. 7B, iv and iv’). In *UAS-dsh/GMR-GAL4; UAS-dve/*+ genotype *Drosophila* there was less reduction in the size of the eyes leading to larger eyes in 100% of the progeny. However, these flies exhibited ommatidial fusion and loss of bristles thus resulting in a rough eye phenotype in all the progeny (Fig. 7B, compare panels v and v’ with iv and iv’). Thus, in a manner similar to *SATB1*, overexpression of *dve* is also sufficient to suppress the gain of function phenotypes of Wnt/Wg signaling components such as *dsh*.

### Ectopic expression of DVE at the D/V boundary of the wing imaginal disc but not that of SATB1 restricts Wg expression

**Figure 8:**
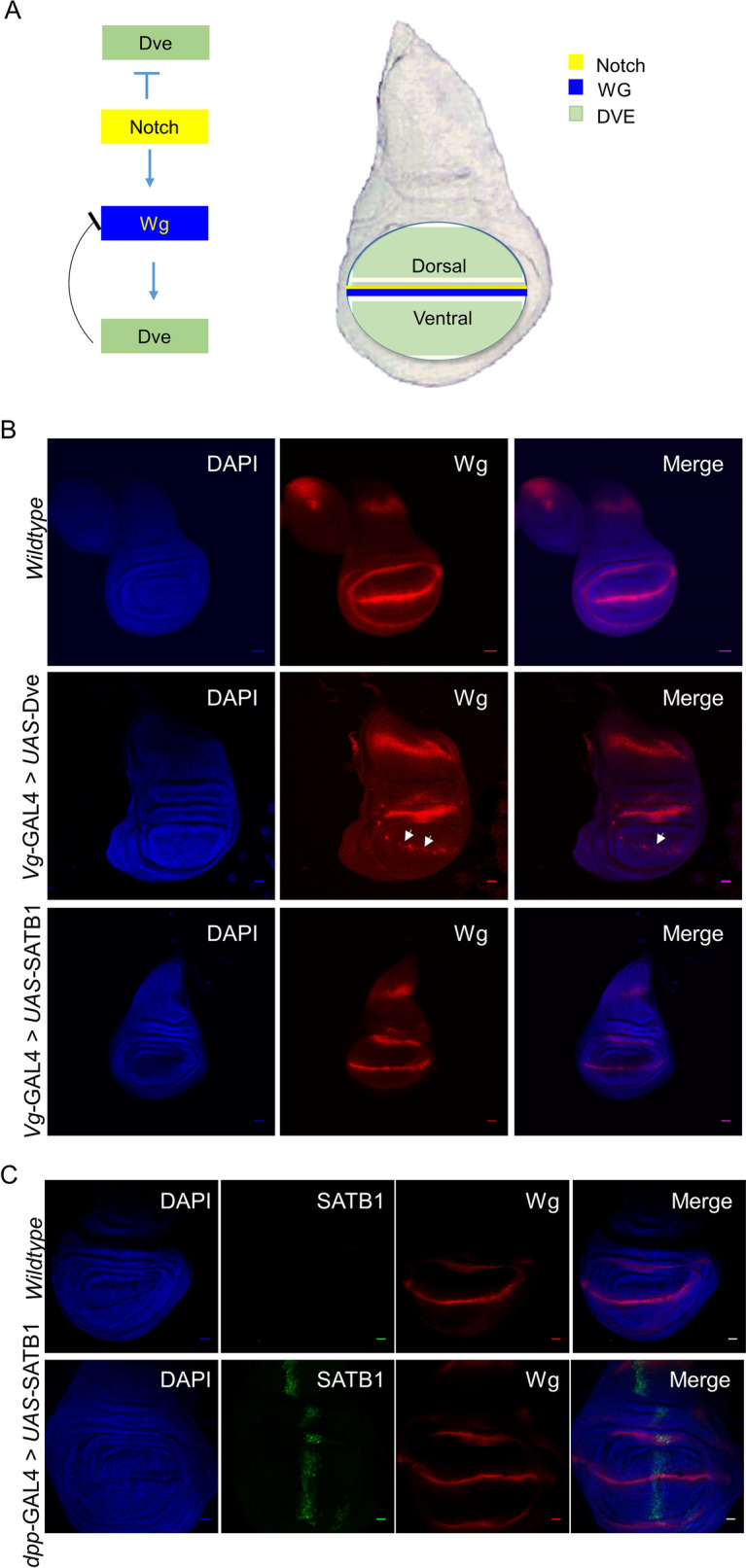
DVE and SATB1 affect Wg expression differently in the wing imaginal disc. (**A**) Schematic representation of the location and inter-relationship of DVE, Notch and Wg in third instar wing imaginal disc. (**B**) Immunofluorescence images of third instar imaginal discs depicting DNA (blue) and Wg expression (red) along the DV boundary in wild type (Top Panel), *vg-GAL4* > *UAS-dve* (Middle Panel), *vg-GAL4* > *UAS-SATB1* (Bottom panel) larvae respectively. White arrows point to disruptions in the pattern of wingless protein at the DV boundary in the wing pouch. (**C**) Immunofluorescence images of wing imaginal discs depicting DNA (blue), Wg expression (red), and SATBI expression (green) from wild type (Top panel) and *dpp-GAL4* > *UAS-SATB1* (bottom panel) larvae respectively. Ectopic expression of SATB1 (green) along the AP boundary did not cause any effect on the pattern of Wg expression (red) at the DV boundary. All images were acquired at a magnification of 25 ×. Scale bars represent 20 μm.

During wing development, DVE is involved in pattern formation along the proximal distal axis (PD) and proliferation of the wing pouch cells^30^. Expression of *dve* is initiated during the early phase of the third instar larval stage, which is dependent on Dpp and Wg signals. As the development of the wing progresses, the regions in which DVE and Wg are expressed become mutually exclusive. In wing imaginal discs of third instar larvae, DVE is expressed in the entire wing pouch with the exclusion of the dorsoventral boundary, whereas Wg is expressed specifically at the DV boundary^30^. When *vg*-*GAL4* was used to ectopically express DVE along the DV boundary of the wing imaginal disc, the pattern of Wg staining along the DV boundary was severely disrupted (Fig. 8B). This finding correlates with previously published data^30^. Upon *vg-GAL4-*induced ectopic expression of SATB1 along the DV boundary, the expression of Wg protein was not affected (Fig. 8B). Additionally, SATB1 was also expressed along the anteroposterior (AP) boundary of the wing disc using *dpp-GAL4*. Upon expression of SATB1 along the AP boundary, no interruption in the expression of Wg protein was observed as revealed by immunofluorescence (Fig. 8C). These results suggest that expression of SATB1 does not affect the levels of Wg protein; however, it may alter the levels of the downstream components of the Wnt/Wg signaling cascade. The discrepancy in the disparate effect on levels of WG can be attributed to the fact that the wing pouch of the disc contains high levels of DVE but has no SATB1 (Fig. 8A).

### SATB1 and DVE have distinct molecular targets

**Figure 9:**
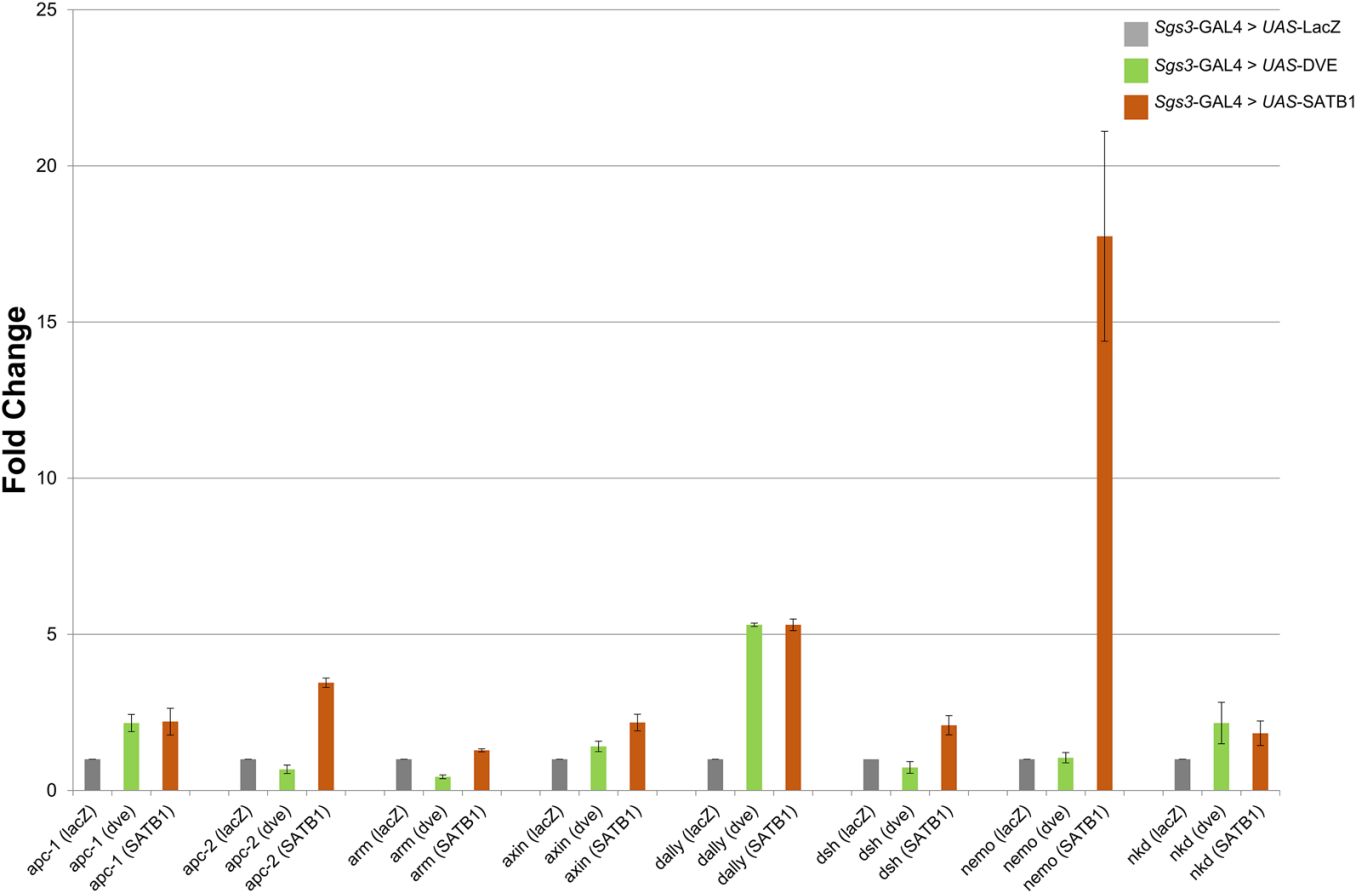
SATB1 and DVE have differential molecular targets. Transcript levels of Wnt responsive genes and Wnt antagonists were estimated by quantitative real time PCR in eye imaginal discs from third instar larvae expressing *Sgs3-Gal4* > *UAS-dve* (green)*, Sgs3-Gal4* > *UAS-SATB1* (red), and *Sgs3-Gal4* > *UAS-lacZ* (grey) control*.* Four genes exhibit maximal differential expression, *apc-2*, *arm*, *dsh,* and *nemo*. Expression of *dsh* exhibits a mild increase upon ectopic expression of SATB1 whereas overexpression of *dve* does not result in any increase. Rp49 was used as an internal control. Error bars indicate standard deviation calculated from triplicates (N = 3). Fold change as compared to control is depicted on Y-axis.

Even though the expression of DVE and human SATB1 result in similar phenotypes in some tissues, the molecular basis underlying these phenotypes and the genes regulated may be different. To comparatively analyze the effects of SATB1/DVE ectopic expression/over-expression on expression of genes involved in the regulation of Wnt/Wg signaling pathway, the transcript levels of Wnt-responsive genes and Wnt antagonists were quantified by qRT-PCR. This experiment was performed using RNA isolated from the salivary glands of third instar larvae since the level of *dve* RNA in the salivary glands is very basal (undetectable by quantitative RT-PCR), thus the larval salivary glands in transgenic flies would mimic a condition where both SATB1 and DVE are ectopically expressed. The expression of SATB1 and DVE was induced using *Sgs3-GAL4* as salivary glands lack any endogenous expression of DVE. Four genes namely *apc-2*, *arm*, *dsh*, and *nemo* exhibited maximal differential expression upon ectopic expression of SATB1. Levels of *dsh* transcripts increased by twofold upon ectopic expression of SATB1 but did not exhibit any significant increase in response to ectopic expression of *dve.* In contrast, the levels *arm* transcript did not change in response to SATB1 expression and decreased upon expression *dve* in the salivary glands. Transcript levels of *nemo* increased by more than 15-fold, while that of apc-2 increased by twofold in response to SATB1. The ectopic expression of DVE did not lead to any significant change in the transcript levels of *apc, dsh* and *nemo.* These results suggest that SATB1 and DVE may regulate different components of the Wnt/Wg signaling cascade (Fig. 9).

## Discussion

Signaling pathways allow cells to respond to their environment by regulating expression of target genes. The transcriptional control function of each pathway is carried out by one or more signal-regulated transcription factors, which bind to specific regulatory elements called signaling pathway response elements (SPREs) and recruit co-activators and chromatin-remodeling machinery, thereby modulating target gene expression^36,37^. Disproportionate expression of transcription factors that respond to signal cues is thought to re-establish developmental programs out of context, thereby contributing to development and progression of cancer. Special AT-rich binding protein 1 (SATB1) is a nuclear factor that functions as a global chromatin organizer regulating chromatin structure and gene expression^9^. Expression of SATB1 is altered in various types of cancers and may be involved in promoting metastasis^38,39^. SATB1 provides a key link between DNA loop organization, chromatin modification/remodeling and association of transcription factors at matrix attachment regions (MARs). These functions of SATB1 require the C-terminal DNA-binding domain and the N-terminal protein interacting region respectively. The N-terminal half of SATB1 mediates its homodimerization which is a prerequisite for DNA-binding^4,9^.

Mammalian two-hybrid assay was performed to test whether Dishevelled-1 (Dvl) interacts with SATB1. Dishevelled-1 is an upstream regulator of the Wnt/Wg signaling pathway, and is crucial for development, cell fate decisions, proliferation, cell–cell communication, organogenesis, and stem cell renewal^40,41^. The results of the co-immunoprecipitation assay demonstrated that SATB1 and Dvl might be part of a single complex. Wnt/Wg pathway Dishevelled is known to localize to LRP6 signalosome in response to Wnt/Wg signal, a step essential for signal transduction by Wnt^42^. Overexpression of Dishevelled is leads to activation of Wnt pathway^7^ and perturbance in the levels of Dishevelled manifests in the form of abnormalities^43^. Even though Dishevelled doesn’t have a DNA-binding domain it is known to bind and regulate Wnt-responsive genes. It can associate with both c-Jun and β-catenin inside the nucleus, which in turn bind TCF7L2. Knockdown of Dvl disrupts the association of β-catenin:TCF7L2 complex on promoters of Wnt target genes (e.g. *c-myc* promoter) in vivo, leading to altered expression of Wnt responsive genes^44^. Dishevelled (Dvl) contains multiple domains crucial for mediating protein interactions between proteins containing DIX and/or other PDZ domains. This enables the functions of these proteins as scaffolds and enables recruitment of multiple transcription activators onto the genomic loci. Previous studies using thymocytes demonstrated that SATB1 recruits and binds to β-catenin, which is the final effector of the Wnt/Wg pathway, onto the promoters of Wnt-responsive genes. This ultimately leads to altered transcriptional activity of the target Wnt-responsive genes in response to Wnt signaling^3,45^. Furthermore, SATB1 also competes with TCF7L2 for binding to β-catenin^3,45^. Colorectal cancers which exhibit elevated Wnt/Wg signaling also express SATB1 at higher levels^3,30,46^. Based on these observations we sought to determine whether alteration in SATB1 expression has any effect on Wnt signaling and expression of Wnt responsive genes. SATB1 overexpression led to upregulation of multiple bonafide Wnt-responsive genes such as CtBP1, TCF7L2, c-fos (Fra-1), c-jun, c-myc, and cyclin D1 (CCND4), and repression of Wnt-antagonistic genes such as Nkd, Dkk, and SFRP4 at transcript level. These results were similar to those observed upon Wnt3A stimulation. Transcript levels of Dvl-1 were also enhanced upon SATB1 expression suggesting that SATB1 overexpression indirectly activates the Wnt pathway in thymocytes. Study of Wnt3A-mediated stimulation of HEK293 cells revealed two phases of transcriptional regulation: 1) an early phase in which early Wnt-responsive genes such as c-myc and CCND1 are upregulated, and signaling antagonists such as Dkk, Nkd and Axin are held in check (0–3 h), and 2) a later phase in which many of these same antagonists are upregulated (3–24 h), attenuating the signal^47^. In contrast, a significant difference in transcript levels of the antagonists was not observed between the control cells and those treated with Wnt3A at 3h^47^. Overexpression of Dishevelled-1 led to a slight decrease in transcripts of Nkd2 and SFRP4. These results are also in agreement with the earlier report that SATB1 positively regulates the Wnt signaling pathway in colorectal cancer cells^48^. However, these results are contradictory to the regulation in thymocytes^3,46^ wherein an inverse correlation was observed between SATB1 and c-myc, a Wnt-responsive gene. Thus, regulation of the Wnt/Wg pathway by SATB1 is a context-dependent phenomenon which warrants further investigations. We used the approach of ectopic expression of mammalian SATB1 in a system that lacks endogenous SATB1 and has a fully functional Wnt/Wg pathway to investigate if regulation of Wnt/Wg pathway is a conserved phenomenon or if it is context-dependent. Although *Drosophila* does not express an identified homolog of human SATB1, it expresses conserved homologs of various known SATB1 interacting factors such as Arm and Dsh, and most of the components of the chromatin machinery.

Heterologous expression of the human genomic organizer SATB1 in *Drosophila* under the control of *GMR-GAL4* driver led to generation of a rough eye phenotype due to fused ommatidia and improperly organized bristles. Immunostaining of SATB1 in the eye revealed that ectopically-expressed SATB1 resided entirely within the nuclei and excluded the DAPI-rich regions, similar to the SATB1 staining pattern observed in thymocytes^3,46^. This suggests that the cellular machinery required for proper processing and transport of the SATB1 protein to the nucleus is functional in *Drosophila*. Ectopic expression of the N-terminal SATB1 (1-204) gave rise to mild phenotypes, whereas ectopic expression of the DNA-binding region (255-763) alone did not result in a visible phenotype. Thus, in an in vivo system, in addition to the N-terminal protein interaction interface, the DNA- binding property of SATB1 also plays a significant role in SATB1 function. However, we cannot entirely rule out the possibility that SATB1-(1-204) and SATB1-(255-763) might be less stably expressed than full length SATB1 protein, resulting in the absence of any strong phenotypes upon their ectopic expression in *Drosophila*. Experiments performed in cell lines pointed towards a positive correlation between the Wnt/Wg signaling pathway and SATB1. To validate if the positive correlation observed between SATB1 and the Wnt/Wg pathway in cell-lines holds true in vivo, SATB1 was overexpressed in the Wnt activation background in *Drosophila*. Overexpression of Wnt pathway intermediaries such as *dsh* or *arm* in the fly eye leads to a small eye phenotype wherein all the ommatidia are completely fused together. Simultaneous expression of both SATB1 and Dvl or Arm in the *Drosophila* eye resulted in partial suppression of the small eye phenotype. This observation suggests a negative correlation between SATB1 and the Wnt/Wg pathway, which is contrary to what was observed in the mammalian cells where it activates the Wnt/Wg pathway. The suppression of the small eye phenotype by SATB1 could be due to sequestration of various proteins that might be involved in the Wnt signaling pathway by SATB1, thereby causing downregulation of Wnt signaling. Another possibility is the ectopic expression of SATB1 may lead to novel protein interactions in *Drosophila* cells which may otherwise be absent and a high concentration of SATB1 may be sufficient to activate genes which are otherwise negatively regulated by Wnt/Wg pathway. To test if the phenotype observed upon SATB1 ectopic expression could be due to binding of SATB1 to the *Drosophila* genome or the result of the misexpressed protein sequestering endogenous *Drosophila* proteins, polytene staining was performed. This analysis revealed at least four strong bands corresponding to SATB1 occupancy which were located near the chromocenter of the polytene chromosome. These regions are known to be highly heterochromatinized with a central block of heterochromatin, surrounded by a large number of smaller, interconnected blocks which further continue into the euchromatic chromosome arms^49^. Thus, this analysis revealed that, mammalian SATB1 could potentially bind *Drosophila* chromatin in vivo*.* However, this result does not rule out a fraction of SATB1 that might work in chromatin-independent manner. The observed phenotype may be due to both binding of ectopically-expressed SATB1 to *Drosophila* genome and sequestration of *Drosophila* proteins.

Genome-wide binding site analysis using ChIP-sequencing of salivary glands from SATB1-expressing transgenic flies could further confirm the binding of SATB1 to these loci and also identify additional binding sites if any. Further, quantitative PCR analyses using eye imaginal discs showed ectopically expressed SATB1 positively regulated the transcript levels of antagonists of the Wnt/Wg pathway, such as *nkd*, *dally*, and *apc-2,* while no significant effect was observed on the transcript levels of positive regulators like *dsh*. Thus, in *Drosophila* ectopically expressed mammalian SATB1 negatively regulates the Wnt/Wg pathway by activating the transcription of the antagonists of the pathway. Presence of consensus SATB1-binding motifs in the promoter regions of these genes was established by performing MEME analysis. It would be interesting to investigate if an endogenous fly protein similar to SATB1 would function in a similar manner and further support our findings.

To this date none of the known *Drosophila* proteins exhibit domain architecture similar to SATB1 i.e. comprising a ubiquitin-like domain (ULD), CUT domain and a homeodomain. Defective proventriculus (DVE), a protein involved in the development of proventriculus has been identified as the protein most similar to the vertebrate SATB1, based on phylogenetic analysis of protein structure^2,27^. Both DVE and SATB belong to the CUT superclass of homeobox genes and have an evolutionarily conserved COMPASS (CMP) domain^2^. As opposed to sequence level homology, these reports are based on structural prediction analysis. To determine functional similarity between these two proteins, the effect of overexpression of *defective proventriculus* (*dve*) on regulation of Wnt/Wg pathway was studied. The homeobox-containing gene *defective proventriculus* (*dve*) is expressed in various tissues including the head primordium and functions as a transcription factor. The ectopic expression of *dve* resulted in a rough eye phenotype, though the intensity of this phenotype was stronger than that observed upon expression of SATB1; this can be attributed to basal levels of DVE present in the eye. Co-expression of *dve* and *dsh* during eye development using the GMR-*GAL4* driver resulted in all progeny having a rough eye phenotype without any reduction in the size of the eye. This observation indicates that DVE is sufficient to suppress the phenotypes associated with Wnt activation. This phenomenon is also observed in other tissues of *Drosophila*. For example, overexpression of *dve* in adult intestinal stem cells of the midgut is sufficient to suppress the phenotype associated with constitutive activation of the Wnt pathway^50^. In the wing disc, DVE is expressed in the wing pouch with the exclusion of the DV boundary. While expression of DVE along the DV boundary resulted in restriction of Wingless (Wg) expression, SATB1 expression did not yield any such an effect. Thus, even though ectopic expression of DVE and SATB1 give rise to similar phenotypes in some tissues, the underlying molecular mechanisms might be different. The results from these studies suggest that, DVE and SATB1 might have few common and many distinct functions. Incidentally, the *C. elegans dve* (*dve-1*), is known to function in a manner similar to human SATB1 in determining lifespan^51^. Therefore, we propose that *Drosophila* DVE might be an ancestral molecule which may have evolved to give rise to SATB1 in the vertebrates. This hypothesis is corroborated by studies of Burglin and Cassata that proposed a connection between the evolution of metazoans and the early COMPASS/CUT genes to the SATB family genes in vertebrates^2^. It is unclear if SATB family genes evolved due to gain of sequences by the vertebrate lineages or loss from other phyla^2^. In a parallel branching, the primordial COMPASS/CUT gene lost its CUT domain and duplicated its homeodomain. Thus, vertebrate SATB1 and *Drosophila* DVE are proteins with partly overlapping activities that might have evolved from a common ancestral protein while their functions subsequently diversified during the course of evolution.

## Methods

### Cell lines and reagents

HEK293 cells were grown in Dulbecco’s Modified Eagle’s Medium (DMEM, Life Technologies, Carlsbad, CA, USA) supplemented with 10% fetal bovine serum (FBS, Life Technologies, Carlsbad, CA, USA), under 5% CO_2_ atmosphere. pTriEx-3 Neo vector was procured from Novagen/EMD Biosciences (CA, USA). Cells were transfected with pTriEx SATB1 and pTriEx Dishevelled-1 (Dvl-1) constructs using Lipofectamine 2000 (Life Technologies, Carlsbad, CA, USA). DNA to Lipofectamine ratio was used as per manufacturer’s instructions. Recombinant human Wnt3A was procured from R&D Systems (Minneapolis, MN).

### Mammalian two-hybrid assay

CheckMate mammalian two hybrid system (Promega Corp., USA) was utilized to score for protein–protein interactions. This system comprises three vectors, (1) pBIND, containing the yeast GAL4 DNA-binding domain (DBD) upstream of the multiple cloning site, (2) pACT, containing the herpes simplex virus VP16 activation domain upstream of the multiple cloning region, and (3) pG5luc reporter, containing five GAL4-binding sites upstream of a minimal TATA box, which in turn is placed upstream of the firefly luciferase gene. The firefly luciferase reporter gene is expressed when the DNA-binding domain in the pBIND vector and the transcriptional activation domain in the pACT vector come into contact via the interaction between the prey and bait proteins (Fig. 1A). The cDNA sequences encoding potential interactive proteins (prey and bait) were cloned into the pBIND and pACT vectors to generate GAL4 and VP16 fusion proteins respectively (Fig. 1B). The pGAL4 and pVP16 fusion constructs were transfected along with the pG5luc reporter into HEK293 cells. Twenty-four hours prior to transfection, 5 × 10^4^ HEK293 cells were seeded per well in a 24-well plate (BD Falcon). HEK293 cells were grown up to 70% confluence in 24-well plates at 37 °C in DMEM supplemented with 10% FBS in an atmosphere of 5% CO_2_. Transfections were performed using pG5luc reporter along with pBIND fusion construct and pACT empty vector in control and pBIND fusion construct with pACT fusion construct for the experimental set. DNA was transfected using Lipofectamine 2000 (Life Technologies, Carlsbad, CA, USA), as per the manufacturer’s instructions, in serum-free medium. Total DNA per well was maintained constant at 1.5 μg (0.5 μg of each DNA). Cells were harvested 48 h post-transfection. The medium was supplemented with 10% fetal bovine serum and 0.5 μL/mL enhancer solution (Perkin Elmer, Shelton, CT, USA), 6 h post-transfection. Transactivation assays were performed 48 h post-transfection. Cells were harvested, washed with buffer (1x PBS containing 1 mM CaCl_2_ and 1 mM MgCl_2_) and resuspended in 25 μL of buffer (1x PBS containing 1 mM CaCl_2_ and 1 mM MgCl_2_), equal volume of Steadylite Plus reagent (Perkin Elmer, Shelton, CT, USA) was added and the resultant chemiluminescence was measured on TopCount-NXT Microplate Scintillation Counter (Perkin Elmer, Waltham, MA, USA). Fold changes were calculated by normalizing the transfected sample values to the vector control values.

### Co-immunoprecipitation assay

HEK293 cells were grown to 70% confluency in 90 mm culture dishes. Ten micrograms of purified plasmid was used for transfection per 90 mm culture dish. Transfections were performed as per the manufacturer’s instructions, and cells were harvested 48 h post-transfection. Cells were lysed in RIPA buffer (150 mM NaCl, 0.1% sodium dodecyl sulphate, 10 mM sodium phosphate, 1% sodium deoxycholate, 2 mM EDTA [pH 8.0], 1x EDTA-free complete protease inhibitors). The lysate was diluted to a final concentration of 1 μg/μL using 1x chilled PBS containing 1x EDTA-free complete protease inhibitor cocktail (Roche, Indianapolis, IN, USA). For each immunoprecipitation reaction, 600 μg of the lysate was precleared for 1 h at 4 °C on an end-to-end rocker with 10 μL protein A/G Plus UltraLink resin (Thermo Scientific, Rockford, IL, USA). Post pre-clearing, the supernatant was collected by centrifugation at 1000 × *g*, 5 min at 4 °C. Pre-cleared extract was incubated with 1 μg of antibody for 4 h at 4 °C on an end- to-end rocker. This complex was then immunoprecipitated using 10 μL of protein A/G beads. These protein A/G resin-bound protein-antibody complexes were recovered by centrifugation at 1000 × *g* for 5 min. Beads were washed five times with PBS containing 0.1% Triton X-100. The complexes were eluted by boiling the beads in 30 μL Laemmli sample buffer (with DTT) at 95 °C for 5 min with intermittent mixing and eluate was resolved on a 10% SDS-PAGE gel and transferred to PVDF membrane (Millipore). Immunoblotting was performed using an antibody against the second protein, followed by incubation with horseradish peroxidase (HRP)-conjugated anti-IgG antibody. Signal for immunoblot was detected using Visualizer Western blot detection kit (Millipore/Upstate, Billerica, MA, USA).

### RNA extraction and quantitative PCR

Total cellular RNA was extracted using TRIzol reagent (Life Technologies). Isolated RNA was then subjected to DNase treatment (Promega) as per the manufacturer’s instructions. RNA was further purified using acid phenol: chloroform, followed by ethanol precipitation. One microgram of purified RNA was reverse transcribed into first strand cDNA using ImProm-II Reverse Transcription System (Promega). The resulting cDNA was subjected to qPCR using Power Sybr reagent (ABI) with specific set of primers, as described previously^48^. Changes in threshold cycle (Ct) values were calculated as follows: ∆Ct = (Ct_target genes_ – Ct_internal control_) for transcript analysis. These ∆Ct values were used to calculate fold change using an equation where relative fold change = 2^-(∆(∆Ct))^_,_ and graphs were plotted for average fold values with standard deviation from three independent experimental samples using Sigma Plot.

### Fly culture and stocks

All experiments were carried out at 25 °C on standard cornmeal/molasses/yeast/agar medium (corn-flour 75 g, sugar 80 g, yeast 24 g, agar 10 g and malt 60 g per 1000 mL), propionic acid (5 mL/L), ortho-phosphoric acid (1 mL/L) and p-methyl benzoate (5% solution/L) were added prior to pouring the media.

### GAL4 drivers

*GMR-GAL4*^52^, an eye-specific GAL4 whose expression is restricted to the presumptive photoreceptor cells posterior to the morphogenetic furrow in the eye imaginal disc; and *Sgs3-GAL4* (BL6870; w[1118]; P{w[+ mC] = Sgs3-GAL4.PD}2AATP1) expressed in the salivary glands of wandering third instar larvae were used. *dpp-GAL*^*440.6*^ driver^53^, was used to drive expression along the anteroposterior boundary (AP) in wing imaginal discs; *vg-GAL4* driver^54^ was used to drive expression along the dorsoventral compartment boundary of the wing imaginal discs.

### UAS lines for ectopic expression

*UAS-dve* (BL7086)*; UAS-arm.S10,* the Arm^S10^ mutant has a deletion of 54 amino acids in its N-terminal domain, by virtue of which it remains constitutively active^55^; *UAS-dsh*. *UAS-SATB1, UAS-FLAG-SATB1 (1-204),* and *UAS-SATB1 (255-763)* were generated in-house.

### Preparation of polytene chromosome spreads

Polytene spreads were prepared as per the specifications of the protocol from the Cavalli laboratory^56^. Briefly, third instar larvae were collected and the salivary glands dissected in solution 1 (0.1% Triton X-100 in 1x PBS; pH 7.5), care was taken to remove fat body cells and finish dissection within 20 min. Glands were transferred onto a poly-L-lysine coated slide. Here, a drop of freshly prepared solution 2 (3.7% Paraformaldehyde, 1% Triton X-100 in 1x PBS; pH 7.5) was added and the glands fixed for 30 s, these were then moved to freshly made solution 3 (3.7% Paraformaldehyde and 50% acetic acid in 1x PBS; pH 7.5) and incubated for 30 s. Glands were then washed with 1x PBS to remove excess fixative. A coverslip was placed over the salivary glands. Using a pencil, uniform pressure was applied on the salivary glands, avoiding lateral movement of the coverslip. Excess liquid was blotted off and the slide frozen in liquid nitrogen. The coverslip was flicked off using a needle. Slides were washed twice with 1x PBS, 15 min each, and processed for immunostaining.

### Immunostaining of polytene chromosomes

Slides were washed 2 × 15 min in 1x PBS, blocked at room temperature for 1 h in blocking solution (3% BSA, 0.2% (w/v) NP-40, 0.2% Tween 20, 10% non-fat dry milk in 1x PBS; pH 7.5). Polytene spreads were incubated at room temperature for 4 h with 50 µL of SATB1 antibody (In-house) (1:30 dilution in blocking solution) inside a humidified chamber. Slides were washed twice with wash buffer (400 mM NaCl, 0.2% NP-40, 0.2% Tween 20 in 10 mM Phosphate buffer; pH 7.5). Slides were rinsed using 1x PBS and incubated for 1 h at room temperature with 50 µL of fluorescently-labeled secondary antibody (Molecular Probes, Invitrogen) prepared in blocking solution at a dilution of 1:200. Slides were washed for 15 min with wash buffer. DAPI (SIGMA) was used at a working concentration of 1 µg/mL to stain the DNA. Slides were washed twice for 15 min in wash solution and once using 1x PBS for 10 min. A drop of mounting medium (DAKO-cytomation) was placed over the polytene spread and a coverslip was carefully placed over the same. Excess liquid was blotted away and the slide acquired for imaging.

### Immunostaining of imaginal discs

Larvae were collected in a cavity block, washed and dissected in 1x PBS. To access the imaginal discs, a cut was made at the posterior two third part of the larvae and slight pressure was applied to expose the contents of the larval gut. The excess tissue was removed and the remaining 1/3rd was turned inside out with the help of a pair of needles. The exposed imaginal discs attached to the inverted cuticle were fixed using 4% paraformaldehyde prepared in 1x PBS containing 0.1% Triton X-100 (1x PBST) for 10 min at room temperature. Discs were given three 1-min rinses with 1x PBST followed by three 10 min washes with the same. Wing discs were blocked for 2 h at room temperature in blocking solution (0.5% BSA, 2% FBS in 1x PBST; pH 7.4). Samples were incubated overnight with primary antibody prepared in blocking buffer minus Triton X-100. Discs were washed twice with blocking buffer and incubated for 2 h at room temperature with fluorescently labeled secondary antibody (Molecular Probes, Invitrogen) prepared in blocking solution without FBS. Two 15 min washes with 1x PBST were performed, followed by two 10 min washes with 1x PBS. Discs were incubated for 10 min with DAPI (SIGMA; working concentration, 1 µg/mL). Two 10-min washes were performed using 1x PBS. The desired imaginal discs were detached and mounted in mounting medium (DAKO-cytomation).

### Image acquisition

Confocal immunofluorescence microscopy: The images of antibody-stained polytene chromosomes and imaginal discs were captured using an upright confocal microscope (Carl Zeiss LSM 710) using following objective lenses: Plan Apochromat 25x/NA 0.8 Oil, Plan Apochromat 40x/NA 1.4 Oil, Plan Apochromat 100x/NA 1.4 Oil (all Carl Zeiss), at 23 °C. Secondary antibodies conjugated to two fluorochromes were used, Alexa Fluor 488 (Goat anti-Rabbit) and Alexa Fluor 594 (Goat anti-mouse). The images were acquired using Zen 2010 acquisition software (Carl Zeiss) and further processed using ImageJ (NIH). Only the brightness and contrast were altered for entire image without changing the grey values.

Scanning electron micrograph imaging: One day prior to imaging, flies were collected and stored at 4 °C in a 1.5 mL tube. On the day of imaging, flies were kept on ice. Flies were mounted on the imaging stubs under a dissection microscope using a needle and enough pressure was applied so as to stick the flies to the double-sided tape placed on the stub. Imaging was performed at 10,000 psi. Images were acquired on Scanning electron microscope FVO-LS10 (Zeiss) at multiple magnifications, 95 ×, 120 ×, 400 ×, and 750 ×. Using ImageJ, only, the brightness and contrast of the images were altered without changing the grey values.

## Supplementary Information

Supplementary Figures.
