# Supplementary material for: Global chromatin organizer SATB1 acts as a context-dependent regulator of the Wnt/Wg target genes
Source: Sci Rep. 2021 Feb 9;11:3385. doi: 10.1038/s41598-021-81324-2 (PMC7873079; doi:10.1038/s41598-021-81324-2)
Supplement: Supplementary file 1 — Supplementary Figures. [file 41598_2021_81324_MOESM1_ESM.pdf]

Global chromatin organizer SATB1 acts as a context-dependent regulator of the Wnt/Wg target genes

Praveena L Ramanujam[1,2], Sonam Mehrotra[1,2,3], Ram Parikshan Kumar[4], Shreekant Verma[4], Girish Deshpande[5], Rakesh K. Mishra[4,6], and Sanjeev Galande[1,6]

1- Centre of Excellence in Epigenetics, Department of Biology, Indian Institute of Science Education and Research, Pune 411008, India

2- These authors contributed equally

3- Advanced Centre for Treatment, Research and Education in Cancer, Kharghar, Mumbai, India

4- Centre for Cellular and Molecular Biology, Hyderabad, India

5- Department of Molecular Biology, Princeton University. Princeton NJ 08540, USA

6- Senior authors to whom correspondence should be addressed

Short Running Title: Context-dependent regulation of Wnt/Wg pathway by SATB1

Address for Correspondence:

Sanjeev Galande
Centre of Excellence in Epigenetics
Indian Institute of Science Education and Research
Dr. Homi Bhabha Road, Pune 411008, India
Tel: 91-20-25908060, Fax: 91-20-20251566
email: sanjeev@iiserpune.ac.in

Supplementary Information

Supplementary Figure S1

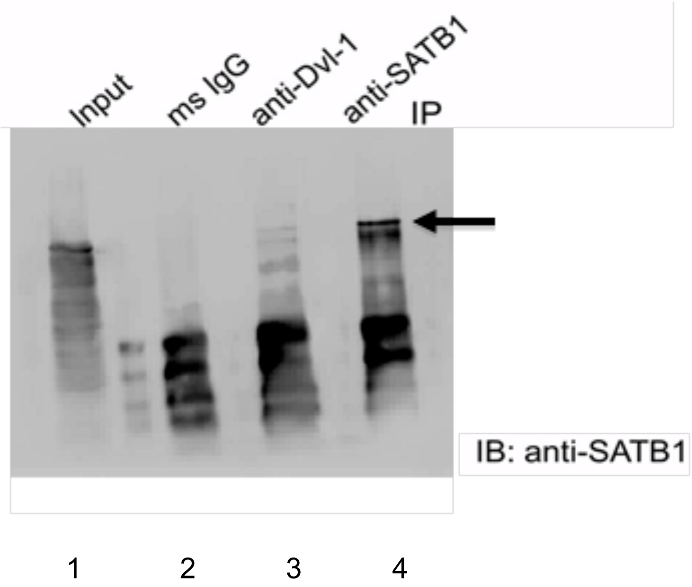

Figure S1: Full blot for the coimmunoprecipitation assay. Lane 1, input (5%). Lane 2, samples immunoprecipated using mouse IgG and immunoboltted using the anti-SATB1 antibody. The signal between the ms IgG sample and input lanes is the overflow from the msIgG sample. Lane 3, samples immunoprecipated using anti-Dvl-1 antibody and immunoboltted using the anti-SATB1 antibody. Lane 4 represents the positive control, wherein the samples immunoprecipated using the anti-SATB1 antibody were immunoboltted using the anti-SATB1 antibody.

Supplementary Figure S2

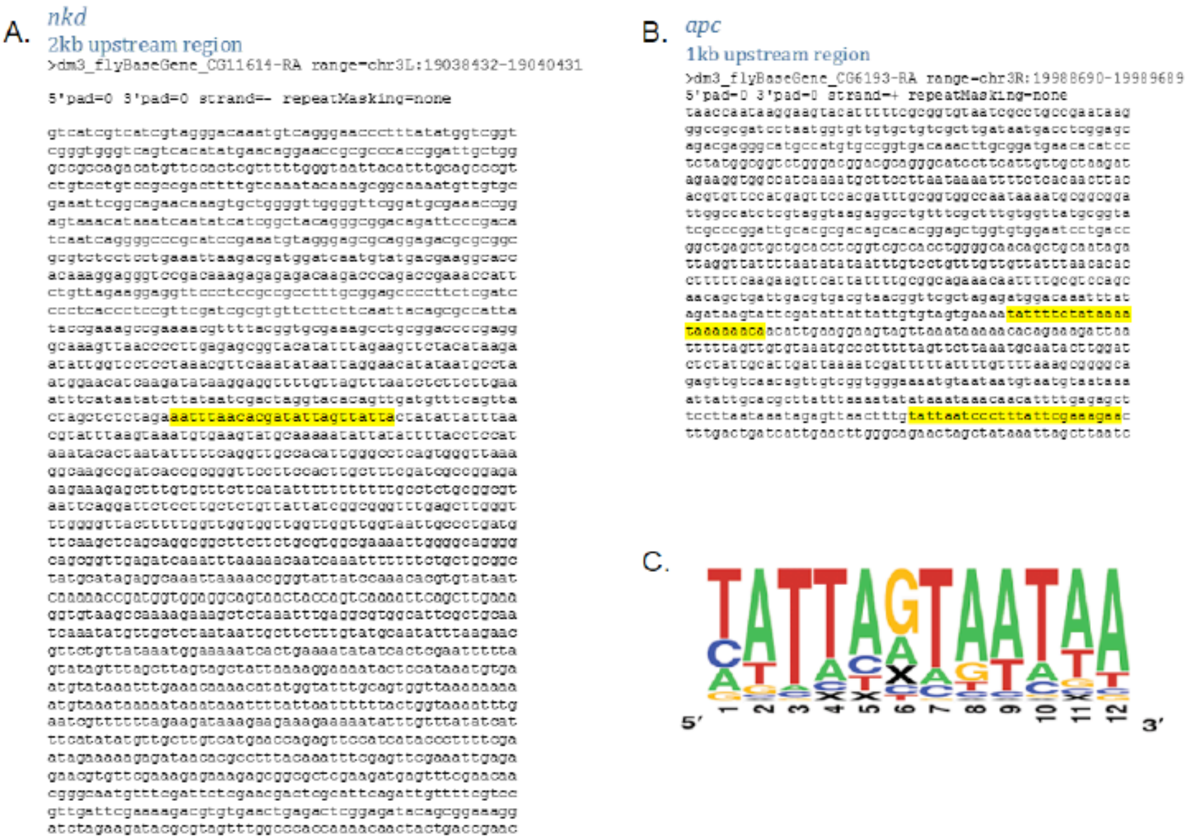

Figure S2: SATB1-binding sites are present upstream to the TSS of *naked cuticle* (*nkd*) and *apc-2*. The sequences upstream sequences of *naked cuticle* and *apc-2* genes were analyzed for the presence of consensus SATB1 binding sites (CSBS) by motif search analysis using MEME. CSBS (C) was identified in the region upstream to the TSS of *nkd* (A) and *apc-2* (B) genes (highlighted in yellow). The upstream regulatory region of *apc-2* gene shows presence of two CSBS sites (B). Hence, mammalian SATB1 can potentially bind these sequences *in vivo*.

Supplementary Figure S3

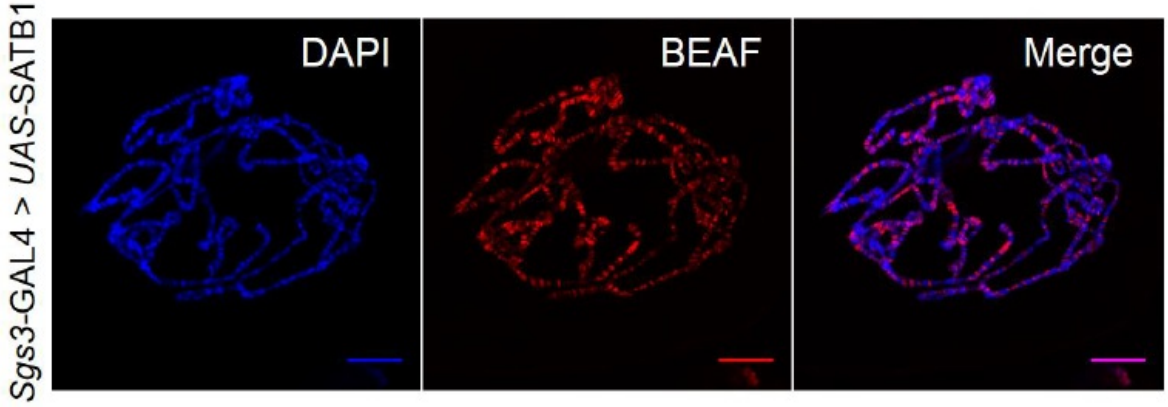

Figure S3: Immunostaining of polytene chromosomes for *Drosophila* matrix binding protein BEAF. Polytene chromosome spreads were prepared from salivary glands of third instar larvae expressing human SATB1 under the control of *Sgs3-GAL4* as described in 'Methods'. Antibody for BEAF, a boundary associated MAR-binding protein was used as a positive control for polytene immunostaining depicted in Figure 6. Image was acquired at 63X magnification; the scale bar represents 20 µm.

Supplementary Figure S4

Chromosome	start	stop	score	p-value	q-value	matched sequence	Gene	Function
chr2L	2446260	2446274	20.3	1.13E-07	0.778	CCAATTAGTAATAAA	Decapentaplagic	Development
chr3R	10583595	10583581	21.9	3.69E-08	0.604	CCAATTACTAATAAC	Homothorax	Development
chr2L	8437902	8437888	20.3	1.15E-07	0.778	CCTATTCGTAATCAC	Chrac-14	Chromatin remodeling
chr3R	17507940	17507954	21	6.65E-08	0.618	CCAATTCGTAATAAC	Down syndrome cell adhesion molecule 3	Cell adhesion
chr2L	12649645	12649631	22.6	1.64E-08	0.648	CCTATTAGTAATAAA	RNA and export factor binding protein 2 (*ref2*)	RNA Export
chr2R	9115437	9115451	21.6	4.71E-08	0.574	CTAATTAGTAATAAC	Phosphorylated adaptor for RNA export (*phax*)	RNA Export
chr3L	5707345	5707359	22	2.83E-08	0.596	CCTATTTGTAATAAC	Still life (*sif*)	Cytoskeletal organization
chr3R	355583	355597	20.9	7.34E-08	0.618	CCTATAACTAATAAC	Myosin 81F	Motor activity
chr3R	25675306	25675320	20.7	8.42E-08	0.618	CCTATTCGTAATAAA	Igr3	GPCR pathway
chr2L	2132094	2132080	20.6	9.99E-08	0.778	CTTATTACTAAAAAC	Glycogen phosphorylase (*glyP*)	Glycogen catabolic process
chr3R	26417202	26417188	21.6	4.71E-08	0.604	CTTTTTAGTAATAAC	CG5432	Glycolysis
chr3R	17425231	17425245	22.6	1.64E-08	0.42	CCTATTAGTAATAAA	CG5873	Response to oxidative stress
chr2R	14095239	14095225	19.7	1.74E-07	0.902	CCAATTTGTAATAAC	Prosap	Scaffold protein (PSD)
chr2R	15176434	15176420	20.3	1.13E-07	0.902	CCTTTTAGTAATAAA	igloo (*igl*)	Neuronal phosphoprotein
chr3L	15855480	15855466	20.7	8.42E-08	0.806	CTTATTTGTAATAAC	pHCl	Chloride channel
chr3R	22847768	22847782	22.6	1.64E-08	0.42	CCTATTAGTAATAAA	CG4704	Unknown
chr2R	19690061	19690047	19.7	1.74E-07	0.902	CCAATTTGTAATAAC	CG15126	Unknown
chr2L	15360959	15360945	20.3	1.13E-07	0.778	CCTTTTAGTAATAAA	CG15258	Unknown

Figure S4: List of putative SATB1-binding sites in *Drosophila* genome. Motif search using MEME analysis identified 21 putative consensus SATB1 binding sites (CSBS) in flies. These sites were highly significant and spread across chromosomes 2 and 3. These binding sites were present in genes involved in varied functions such as chromatin remodeling, metabolic and developmental pathways. Three out of these 21 binding sites are depicted in Figure S2.
